# Climatic Niche Contraction and Refugial Persistence of an Invasive Tephritid Pest Across the Arabian Peninsula Under Contrasting Emission Scenarios

**DOI:** 10.3390/biology15100814

**Published:** 2026-05-21

**Authors:** Hathal M. Al Dhafer, Amr Mohamed, Wei Zhang, Ioannis Eleftherianos, Nemat O. Keyhani, Mahmoud S. Abdel-Dayem

**Affiliations:** 1 King Saud University Museum of Arthropods (KSMA), Plant Protection Department, College of Food and Agriculture Sciences, King Saud University, P.O. Box 2460, Riyadh 11451, Saudi Arabia; 2 Department of Entomology, Faculty of Science, Cairo University, Giza 12613, Egypt; mamr@sci.cu.edu.eg; 3 State Key Laboratory of Green Pesticide, Guizhou University, Guiyang 550025, China; wzhang9@gzu.edu.cn; 4 School of Biological Sciences, Institute for Global Food Security, Queen’s University Belfast, Belfast BT9 5DL, UK; i.eleftherianos@qub.ac.uk; 5 Department of Biological Sciences, University of Illinois, Chicago, IL 60607, USA; keyhani@uic.edu

**Keywords:** Arabian Peninsula, *Bactrocera zonata*, CMIP6 scenarios, habitat suitability, invasion ecology, MaxEnt, pest risk assessment

## Abstract

The peach fruit fly, *Bactrocera zonata* (Saunders) (Diptera: Tephritidae), is a climate-sensitive agricultural invader that threatens fruit production across the Arabian Peninsula, yet its realized climatic niche and future exposure under warming remain insufficiently resolved. We used Maximum Entropy (MaxEnt) modeling to quantify current and projected habitat suitability across the region (~3.2 million km^2^) under two Shared Socioeconomic Pathway scenarios (SSP1-2.6 and SSP5-8.5) for the 2050s and 2070s, based on 55 spatially filtered occurrence records and seven non-collinear environmental predictors, with sampling bias controlled using a Gaussian kernel density bias file. Model performance was robust, with mean training AUC of 0.922 ± 0.011 (SD) and mean TSS of 0.538 ± 0.115 (SD; range: 0.368–0.692), indicating moderate variability across replicates. Suitability was governed primarily by elevation, mean temperature of the driest quarter (Bio 9), mean diurnal temperature range (Bio 2), and precipitation of the coldest quarter (Bio 19), which together contributed over 97% of the model output, indicating strong climatic and topographic control on range persistence. Under present conditions, 790,714 km^2^, or 28.38% of the study area, was suitable, concentrated in the southwestern highlands of Saudi Arabia and Yemen, the Omani mountain ranges, and coastal fringes of the Arabian Gulf and Gulf of Oman. Future projections showed a consistent net contraction of suitable habitat across all scenarios, from 7.4% under SSP1-2.6 in the 2050s to 28.0% under SSP5-8.5 in the 2070s. In all cases, contraction exceeded expansion, although the eastern Omani highlands remained a potential climatic refugium. These patterns indicate that warming is likely to reorganize rather than uniformly expand suitability, providing a spatial basis for climate-informed biosecurity, surveillance, and regional pest management.

## Simple Summary

The peach fruit fly (*Bactrocera zonata*) is a serious pest of fruit crops in the Arabian Peninsula, but it cannot survive equally well in all places. Using climate-based species distribution models, we examined where the insect can live now and how its range may change in the future under different climate warming scenarios. We found that suitable habitat is mainly limited to coastal areas and cooler highlands, especially in Saudi Arabia, Yemen, and Oman. In the future, warming is expected to shrink suitable areas overall, with the greatest losses under the high-emissions scenario. Some mountain regions in Oman may remain important refuges for the species. These results can help guide pest surveillance, quarantine, and long-term management to protect fruit production in the region.

## 1. Introduction

Climate change is reshaping insect pest distributions by altering development rate, survival, voltinism, phenological synchrony, and the geographic space in which populations can persist [1,2]. These physiological constraints can drive geographic redistribution when climatic thresholds are exceeded or newly met, leading to shifts in range limits, altered population stability, and more frequent outbreaks [3,4]. Such effects are amplified by globalization, where recurrent anthropogenic transport facilitates long-distance dispersal and increases the probability that introduced populations encounter climatically suitable conditions for establishment [5]. Tephritid fruit flies are particularly sensitive to this climate–dispersal coupling because invasion success depends on the alignment of thermal suitability, host availability, and propagule pressure, making them responsive indicators of climate-driven biological change [5,6].

From a theoretical perspective, Species Distribution Models (SDMs) provide a formal framework for linking occurrence data with environmental gradients to infer spatial variation in habitat suitability. These models are grounded in niche theory, in which observed distributions are interpreted as partial realizations of broader environmental tolerances constrained by dispersal and biotic filtering [7]. However, realized distributions often diverge from the fundamental niche because of historical contingency and incomplete access to suitable environments, meaning that SDM outputs should be interpreted as probabilistic representations of environmental suitability rather than deterministic expressions of ecological occupancy [8,9].

Among SDM approaches, Maximum Entropy (MaxEnt) modeling has become a dominant framework for presence-only data because it estimates the most uniform distribution constrained by environmental conditions at known occurrence sites [10]. Its extended formulation incorporates regularization and feature selection to improve transferability and reduce overfitting across complex environmental spaces [11,12]. Nevertheless, model behavior is highly sensitive to background selection, sampling bias, and parameterization, requiring careful calibration to ensure ecological interpretability and robust projection under novel climatic conditions [13,14,15]. These considerations are especially critical in arid and semi-arid systems, where occurrence data are sparse and environmental gradients are strongly nonlinear [16,17]. Moreover, because *B. zonata* is an invasive species unlikely to be at distributional equilibrium within the Arabian Peninsula, incomplete range filling may bias model projections toward conservative estimates of future habitat suitability, a limitation that should be considered when interpreting the projections.

*Bactrocera zonata* (Saunders) (Insecta: Diptera: Tephritidae) represents a high-impact model organism for such analyses owing to its broad host range, strong invasive capacity, and substantial economic impacts on horticultural production systems. The species is a highly polyphagous tephritid fruit fly attacking more than 50 host plant species, including economically important crops such as mango (*Mangifera indica*), guava (*Psidium guajava*), peach (*Prunus persica*), and citrus (*Citrus* spp.), reflecting its capacity to exploit diverse cultivated and semi-natural fruiting hosts. Native to South and Southeast Asia, it has expanded westwards into the Middle East and North Africa, establishing populations across arid and semi-arid regions, including widespread distribution throughout the Arabian Peninsula (e.g., Saudi Arabia, Oman, United Arab Emirates, Yemen), where climatic suitability facilitates persistence and seasonal population growth. Its establishment across adjacent North African systems, including Egypt, further reflects a broader invasion corridor linking the Mediterranean–Arabian agro-ecological continuum. Within invaded regions, it infests a wide diversity of fruit crops, where larval development within fruit pulp leads to direct yield loss, post-harvest degradation, and increased phytosanitary management costs, thereby reinforcing its status as a high-risk quarantine pest of international concern [6,18,19,20].

The Arabian Peninsula provides an exceptionally relevant natural laboratory for examining these processes because it is among the most climatically extreme terrestrial regions on Earth. The region is characterized by hyper-aridity, strong spatial heterogeneity in precipitation, and pronounced thermal stress, with sharp environmental gradients between coastal zones, mountainous regions, and interior desert systems [21]. These gradients create narrow ecological margins in which small climatic shifts can produce large changes in habitat suitability. Moreover, observed and projected warming trends under CMIP6 scenarios indicate continued temperature increases and spatially heterogeneous changes in precipitation, with important implications for biological systems operating near physiological limits [22].

Despite the agricultural and biosecurity importance of *B. zonata*, its climatic niche and potential distribution within the Arabian Peninsula remain insufficiently resolved. Existing global assessments provide broad-scale estimates of invasion risk but fail to capture the fine-scale climatic heterogeneity that governs local establishment in arid environments [23]. Consequently, key ecological questions remain unresolved, including the relative importance of elevation, thermal variability, and seasonal moisture in constraining suitability, as well as the spatial configuration of climatically stable refugia under future climate scenarios [18,21]. In addition, the extent to which future climate change under contrasting emissions pathways (SSP1-2.6 and SSP5-8.5) will restructure habitat suitability across the Peninsula remains poorly quantified, limiting the predictive capacity of current risk assessments and potentially influenced by biotic factors such as host plant availability and anthropogenic dispersal pathways associated with agricultural trade and fruit transport networks.

Accordingly, the present study aims to provide a high-resolution, region-specific SDM assessment of *B. zonata* in the Arabian Peninsula. Specifically, it seeks to (i) characterize the species’ current climatic suitability across the region, (ii) identify the principal environmental drivers associated with its distribution, and (iii) project potential range shifts under mid-century and late-century climate scenarios for SSP1-2.6 and SSP5-8.5. By integrating occurrence data with an established presence-only modeling framework, the study is intended to refine understanding of how climatic constraints structure pest distributions in one of the world’s most environmentally extreme and agriculturally vulnerable regions. In doing so, it aims to provide a biologically grounded basis for anticipating future invasion risk, prioritizing surveillance, and informing climate-sensitive pest management strategies.

## 2. Materials and Methods

### 2.1. Study Region

**Figure 1:**
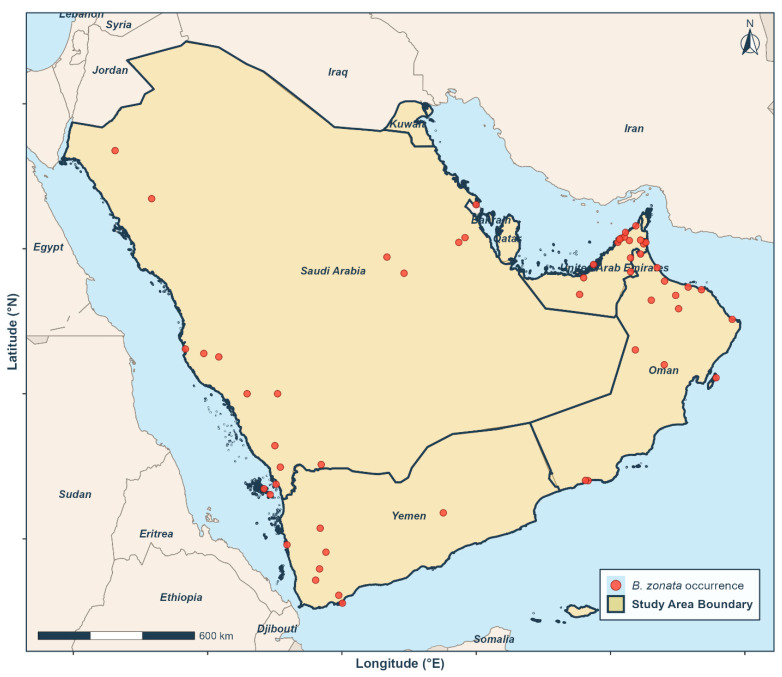
Geographic distribution of confirmed *Bactrocera zonata* occurrence records within the study area across the Arabian Peninsula. Red circles indicate georeferenced locality records used for MaxEnt modeling. The study area (shaded beige) encompasses Saudi Arabia (SAU), Yemen (YEM), Oman (OMN), the United Arab Emirates (ARE), Qatar (QAT), Bahrain (BHR), and Kuwait (KWT).

The study encompassed the Arabian Peninsula and adjacent territories, bounded by approximately 15° N to 33° N latitude and 34° E to 61° E longitude (Figure 1). This domain includes Saudi Arabia, Yemen, Oman, the United Arab Emirates, Bahrain, Qatar, and Kuwait, and covers a total area of approximately 3.2 million km^2^ [24].

The Arabian Peninsula spans a strong climatic gradient, extending from hyper-arid desert interiors, including the Rub’ al Khali (Empty Quarter), one of the world’s largest continuous sand desert systems [25], to comparatively humid coastal margins along the Red Sea, Arabian Gulf, and Gulf of Oman. Mean annual temperatures across the region are generally 25 °C to 35 °C, and summer maxima in inland depressions frequently exceed 45 °C [26]. Precipitation is sparse and highly irregular, averaging less than 100 mm annually across most of the peninsula, with somewhat higher totals in the southwestern highlands of Saudi Arabia and Yemen [27,28].

Georeferenced occurrence records for *B. zonata* used in this study were distributed across the region, with the highest concentrations along the eastern coastal areas of Saudi Arabia, the United Arab Emirates, and Oman, where fruit production is intensive (Figure 1). Additional records were available from inland agricultural areas of Saudi Arabia and from southwestern Yemen, indicating a broad geographic distribution of this pest across the peninsula.

The study region was selected to capture the full range of climatic conditions relevant to the potential distribution of *B. zonata* within the Arabian Peninsula and to provide a comprehensive environmental framework for Maximum Entropy (MaxEnt) species distribution modeling [10,11].

### 2.2. Occurrence Data Collection and Spatial Processing

Georeferenced presence records of *B. zonata* from the Arabian Peninsula were compiled from multiple sources, including the Global Biodiversity Information Facility (GBIF; integrating data from iNaturalist, GenBank/BOLD, and other consolidated databases; GBIF Occurrence Download. Available online: https://doi.org/10.15468/dL.h7vcpw, accessed/revisited on 20 May 2026), the EPPO Global Database, and peer-reviewed publications. Full source details are provided in Supplementary File S1. All records were visually inspected and cross-checked against established distributional information to identify and remove erroneous, duplicate, or spatially uncertain entries. Records lacking reliable geographic coordinates were excluded from further analysis.

A total of 59 occurrence records were initially assembled from seven countries: Saudi Arabia (*n* = 34), the United Arab Emirates (*n* = 15), Oman (*n* = 5), Yemen (*n* = 2), Qatar (*n* = 1), Bahrain (*n* = 1), and Kuwait (*n* = 1). The records spanned the period from 1992 to 2024 and covered an approximate geographic extent of 16.25° N–29.38° N latitude and 36.57° E–56.33° E longitude.

Spatial filtering of the verified dataset was performed with the spThin package [29] in R [30], using a minimum retention distance of 5 km between points. This distance was selected to approximate the spatial resolution of the climate layers (~4 km), thereby removing records that would otherwise contribute redundant environmental information without substantially altering the representation of occupied environmental space [31]. Because the removed records (*n* = 4; 6.8%) were closely proximate to retained points within areas of intensive agricultural monitoring, they were unlikely to represent unique environmental conditions not already captured by neighboring retained occurrences. This procedure preserves spatial independence among occurrences and reduces the influence of spatially autocorrelated records on model calibration [31]. After filtering, 55 records were retained for modeling, representing a reduction of 4 records (6.8%) from the original dataset. All R scripts used in this study are provided in Supplementary File S2.

### 2.3. Sampling Bias Mitigation

Geographic sampling bias is a central concern in species distribution modeling because occurrence records often reflect survey effort rather than true ecological patterns of occurrence [32]. In the Arabian Peninsula, *B. zonata* records are spatially uneven, with strong concentration in areas of intensive fruit cultivation and pest monitoring, particularly the Eastern Province of Saudi Arabia and coastal parts of the United Arab Emirates, whereas inland and non-agricultural areas remain comparatively under-sampled.

To reduce this bias beyond spatial thinning, we applied a bias-adjusted background sampling strategy [32]. Using the ‘Create Bias File’ tool in SDMtoolbox 2.0 [33,34] for ArcGIS 10.7, we generated a Gaussian kernel density surface from the occurrence records to represent sampling intensity across the region. This surface was then supplied to MaxEnt to weight the selection of 10,000 background points probabilistically. By linking background selection probability to the bias surface, the procedure ensures that presences are contrasted with background environments sampled under similar survey conditions, thereby limiting the confounding effects of heterogeneous sampling effort [32]. It should be noted, however, that because occurrence records in the Arabian Peninsula are concentrated in areas of intensive fruit cultivation, the kernel density surface may partly reflect the spatial distribution of agricultural activity rather than true entomological survey effort, which could result in residual spatial bias in model calibration that was not fully corrected by this approach.

### 2.4. Environmental Predictor Variables

The analysis used two classes of environmental predictors: 19 bioclimatic variables and one topographic variable (elevation) (Supplementary File S3). To maintain temporal consistency between the occurrence data (1992–2024) and the environmental predictors, monthly climate grids were obtained from TerraClimate [35] through Google Earth Engine [36]. Monthly maximum temperature, minimum temperature, and total precipitation were downloaded as GeoTIFF files at approximately 4 km spatial resolution and pre-clipped to the Arabian Peninsula.

Long-term monthly climatological means were calculated across the 32-year period, and the 19 standard bioclimatic variables (Bio 1–19) were derived using the terra package in R version 4.4.1. This temporal alignment ensures that the predictors reflect the climatic conditions, including contemporary warming trends, experienced by *B. zonata* populations and supports the pseudo-equilibrium assumptions underlying correlative species distribution models. The resulting bioclimatic layers were then resampled to 30 arc-second (~1 km) resolution to match the elevation data from WorldClim 2.1 [37] and the requirements for future climate projections in MaxEnt. Although this resampling aligned all predictor layers to a common 1 km grid, it should be noted that the original TerraClimate bioclimatic layers were derived from ~4 km source data; resampling to 1 km therefore increases spatial resolution nominally without introducing additional climatic information. Ideally, all predictors would be derived at a consistent native resolution. This mismatch is acknowledged as a potential limitation, particularly for mountain environments where topographic and climatic gradients are steep, and should be considered when interpreting fine-scale suitability patterns in highland areas.

To reduce multicollinearity while retaining ecological interpretability, variable selection was conducted before model fitting. Variance Inflation Factor (VIF) analysis was applied to the initial 20 variables using the usdm package in R, and variables were removed stepwise using a conservative threshold of VIF < 5 (Supplementary File S4) [38,39]. Variables were also screened for pairwise collinearity using Pearson correlation coefficients; all retained predictors showed |r| < 0.7, confirming that no two retained variables were strongly correlated. This procedure systematically eliminated highly correlated predictors that could otherwise distort model coefficients or inflate estimates of variable importance. Seven non-collinear predictors were retained, representing the principal environmental gradients expected to shape the distribution of *B. zonata*.

All environmental layers were clipped to the study boundaries and converted to ASCII format using the terra package in R. The contribution of each predictor was evaluated using two complementary measures: percent contribution, which describes the heuristic role of each variable during model training, and permutation importance, which quantifies the decrease in Area Under the Curve (AUC) after random permutation of variable values [10]. Together, these metrics provide a comprehensive assessment of the environmental factors influencing model predictions [13].

### 2.5. Future Climate Projections

Future projections were based on climate data from five General Circulation Models (GCMs): GFDL-ESM4, IPSL-CM6A-LR, MPI-ESM1-2-HR, MRI-ESM2-0, and UKESM1-0-LL. These models were selected because they constitute the core ensemble recommended by the Inter-Sectoral Impact Model Intercomparison Project (ISIMIP3b) [40] and provide broad coverage of key climate sensitivities represented in CMIP6 [41].

High-resolution datasets (30 arc-seconds) for mid-century (2050; 2040–2060) and late-century (2070; 2060–2080) periods were obtained from WorldClim 2.1 [37]. Projections were generated under two Shared Socioeconomic Pathways (SSPs): SSP1-2.6, representing a stringent low-emission scenario, and SSP5-8.5, representing a high-emission fossil-fueled scenario [42,43].

To reduce uncertainty associated with any single climate model, a multi-model ensemble approach was used [44]. For each SSP and time-period combination, the calibrated species distribution model was projected separately onto the climate layers of each of the five GCMs. Final consensus projections were obtained by calculating the pixel-wise arithmetic mean of the continuous suitability outputs. Full model specifications are presented in Supplementary File S5.

### 2.6. MaxEnt Model Implementation and Optimization

Species distribution modeling was conducted in MaxEnt version 3.4.4 [45], selected for its performance with presence-only data and relatively small sample sizes [8,10], as well as its demonstrated utility in climate change projection studies [46]. MaxEnt estimates habitat suitability by comparing occurrence records with background points and produces continuous prediction surfaces ranging from 0 (unsuitable) to 1 (optimal conditions) [47,48].

A total of 10,000 background points were drawn using the bias file described above, with selection probability proportional to estimated sampling effort through MaxEnt’s ‘bias file’ option. Model complexity was optimized before final fitting with the ENMeval 2.0 package [49] in R [30].

Forty candidate models were evaluated across five feature class (FC) combinations, namely Linear (L), Hinge (H), Linear-Quadratic (LQ), Linear-Quadratic-Hinge (LQH), and Linear-Quadratic-Hinge-Product (LQHP), and eight regularization multiplier (RM) values (0.5, 1.0, 1.5, 2.0, 2.5, 3.0, 3.5, and 4.0). Feature classes define the response curve shapes fitted to environmental predictors, whereas the regularization multiplier controls model complexity and penalizes overfitting [14,50].

Model selection was based on the corrected Akaike Information Criterion (AICc), which balances model fit against complexity relative to sample size to reduce overfitting [51,52,53]. The optimal configuration was FC = LQ (Linear-Quadratic features) with RM = 0.5, which produced the lowest AICc value (AICc = 851.27, ΔAICc = 0, wAIC = 0.90, ncoef = 9), indicating substantially stronger support than all competing candidate models (Supplementary File S6). Although a regularization multiplier of 0.5 is lower than some recommendations for small datasets, its selection here was strictly empirical: it emerged from a systematic evaluation of eight RM values (0.5–4.0) using AICc as the selection criterion. The wAIC value of 0.90 indicates that this configuration received the overwhelming majority of model weight, and the resulting nine-coefficient model is parsimonious relative to the number of occurrence records (*n* = 55). Nonetheless, given the small sample size and the dominant influence of elevation, users should be aware that model predictions at RM = 0.5 may be somewhat more tightly fitted to the training data than models using higher RM values, and response curves should be interpreted accordingly.

### 2.7. Model Performance Assessment

Predictive performance was evaluated using two complementary metrics: the Area Under the Receiver Operating Characteristic Curve (AUC) and the True Skill Statistic (TSS). AUC provides a threshold-independent measure of discrimination between presence and background points, with values >0.7, >0.8, and >0.9 indicating acceptable, good, and excellent performance, respectively [54].

TSS provides a threshold-dependent measure of classification accuracy that incorporates both sensitivity and specificity; values > 0.5 indicate good predictive performance, whereas values > 0.6 are considered very good [55]. Using both threshold-independent (AUC) and threshold-dependent (TSS) metrics provides a robust assessment of model quality and follows established best practice in ecological modeling [56,57].

### 2.8. Threshold Selection and Binary Classification

To assess potential spatial shifts in distribution under future climate scenarios, continuous MaxEnt suitability outputs were converted to binary presence-absence maps. The ‘10th percentile training presence’ threshold was used as the classification cut-off, whereby a grid cell was classified as suitable when its predicted suitability value was equal to or greater than the suitability score at the 10th percentile of all training presence records (i.e., the suitability value below which 10% of occurrence localities fell). The 10th percentile training presence threshold, calculated as the mean across ten replicates, was 0.248 (SD = 0.061) and was used to binarize all current and future suitability maps.

This threshold was selected for two reasons. First, from an agricultural biosecurity perspective, the consequences of failing to identify potentially suitable habitat (false negatives) are greater than those of moderate overprediction (false positives) [58]. Undetected suitable areas may permit unmonitored establishment and subsequent spread of the pest. Second, this conservative threshold maintains high sensitivity while accounting for minor spatial uncertainty in georeferenced records and retaining marginal or transitional habitats that may become increasingly favorable under future climate conditions [59,60].

The resulting binary maps were used to compare current and future distributions under SSP1-2.6 and SSP5-8.5 for 2050 and 2070, enabling quantification of spatial change across four categories: stable presence (suitable under both current and future conditions), expansion (newly suitable), contraction (loss of suitability), and stable absence (unsuitable under both conditions).

### 2.9. Multi-Model Climate Projections and Synthesis

In total, 20 future habitat suitability scenarios were generated by projecting the calibrated MaxEnt model across five GCMs, two SSPs, and two future time horizons. Current potential distributions were derived by averaging the continuous suitability outputs from ten bootstrap replicates.

For future projections, consensus maps were generated for each SSP and time-period combination, for example SSP1-2.6 for 2050, by calculating pixel-wise means of continuous suitability values across all five GCMs. This multi-model ensemble framework reduces inter-model uncertainty inherent in future climate simulations and yields robust spatial predictions while preserving a consistent ecological model structure [61,62].

## 3. Results

### 3.1. Model Performance and Environmental Variables

The MaxEnt model showed consistently strong discriminatory performance across all ten replicates (Figure S1 and File S7). Training AUC ranged from 0.909 to 0.944, with a mean of 0.922 ± 0.011 (SD), whereas test AUC ranged from 0.817 to 0.912, with a mean of 0.872 ± 0.029 (SD). All AUC values, for both training and test data, exceeded the commonly used threshold of 0.7 [10], indicating robust predictive performance and low likelihood of random fit. TSS values ranged from 0.368 to 0.692, with a mean of 0.538 ± 0.115 (SD; File S7). Although two replicates, 5 and 8, fell slightly below the acceptable threshold of 0.5 [55], the ensemble mean remained above this benchmark, supporting adequate discrimination between suitable and unsuitable habitat. The larger variability in TSS relative to the stability of AUC is consistent with expected sensitivity differences among cross-validation partitions, particularly in datasets with limited occurrence records. Together, these metrics indicate that the model is suitable for reconstructing current habitat suitability and projecting future distributions of *B. zonata* across the Arabian Peninsula.

Seven non-collinear predictors were retained in the final model, and their relative influence varied markedly (Table 1 and Figure S2). Elevation (Elev) was the strongest predictor, contributing 43.4% to the model with a permutation importance of 29.8%, indicating that topographic heterogeneity exerts the primary constraint on habitat suitability. Temperature-related variables formed the next most important group. Mean temperature of the driest quarter (Bio 9) contributed 18.8% with a permutation importance of 24.2%, while mean diurnal temperature range (Bio 2) contributed 18.7% with a permutation importance of 20.4%, together highlighting the importance of thermal seasonality and daily temperature fluctuation in delimiting suitable habitat. Precipitation of the coldest quarter (Bio 19) also made a substantial contribution, with 16.0% contribution and 19.1% permutation importance, underscoring the species’ sensitivity to winter moisture availability.

By contrast, mean temperature of the wettest quarter (Bio 8), precipitation seasonality (Bio 15), and isothermality (Bio 3) each contributed little to the model, and together accounted for less than 3.1% of total contribution, suggesting only limited refinement of distributional limits. The close agreement between percentage contribution and permutation importance across predictors supports the stability of variable ranking and suggests that no single predictor disproportionately drove model behavior.

**Figure 2:**
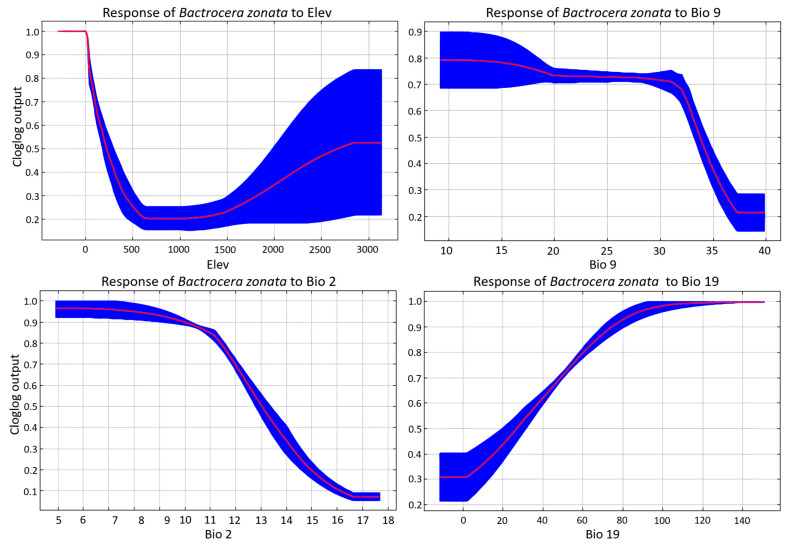
Marginal response curves illustrating the relationship between *Bactrocera zonata* habitat suitability and the four most influential environmental predictors in the MaxEnt model: elevation, Bio 9 (mean temperature of the driest quarter), Bio 2 (mean diurnal temperature range), and Bio 19 (precipitation of the coldest quarter). Red lines represent the mean response across replicate model runs; blue shading denotes ± one standard deviation.

Marginal response curves for the four most influential variables revealed clear and ecologically coherent relationships with habitat suitability (Figure 2). Elevation showed a bimodal, non-linear response. Suitability was highest at or near sea level, declined sharply to a minimum around 500 to 700 m, and then increased progressively above approximately 1500 m, reaching moderate to high values above 2500 m. This pattern indicates that the species occupies both lowland coastal and agricultural zones and higher-elevation highland environments within the Arabian Peninsula, consistent with its association with cultivated fruit-growing areas across altitude gradients.

Bio 9, mean temperature of the driest quarter, maintained high suitability across approximately 10 °C to 28 °C, after which suitability declined sharply and reached minimum values above ~35 °C. This response suggests an upper thermal limit during the driest season and implies that extreme heat constrains establishment in the hottest arid lowlands. Note that the low-temperature side of this response (values below approximately 10 °C) should be interpreted with caution, because it may reflect extrapolation beyond the climatically realized range of the Arabian Peninsula; the model was not calibrated with occurrence records from environments colder than this threshold. Bio 2, mean diurnal temperature range, was negatively related to suitability. Values between 5 °C and 11 °C corresponded to near-maximum suitability, which then declined steeply and almost monotonically beyond 12 °C, approaching minimum suitability above 17 °C. This indicates a preference for thermally stable environments with low day-to-night fluctuation, typical of humid, elevated, or coastal settings.

Bio 19, precipitation of the coldest quarter, showed a strongly positive sigmoidal response. Suitability increased from approximately 0.3 at near-zero precipitation to near-maximum values (≥0.95) above ~80 mm. This pattern indicates a clear preference for areas receiving substantial winter precipitation, likely reflecting the importance of cold-season moisture for host plant productivity and larval development.

### 3.2. Current Habitat Suitability Distribution of Bactrocera zonata

**Figure 3:**
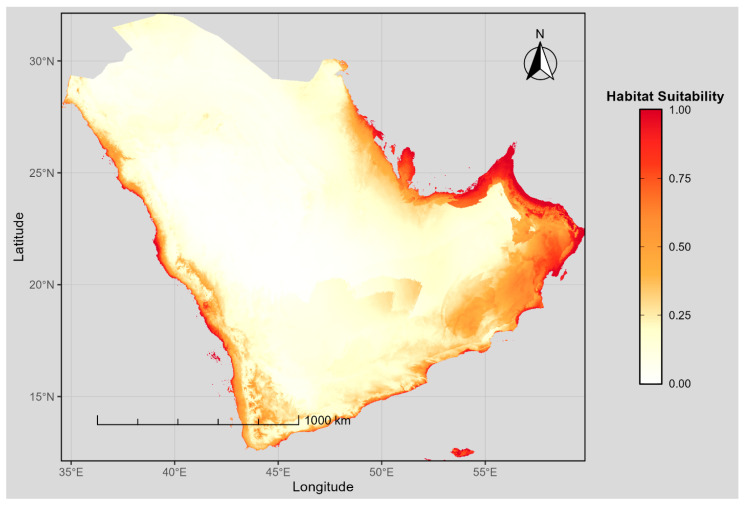
MaxEnt-predicted habitat suitability for *Bactrocera zonata* across the Arabian Peninsula under current climatic conditions. Suitability values range from 0 (unsuitable, white) to 1 (highly suitable, dark red).

Under present climatic conditions, the model estimated that approximately 790,714 km^2^ of the Arabian Peninsula, equivalent to 28.38% of the effective modeled area (approximately 2.79 million km^2^ of terrestrial land surface, after exclusion of water bodies and areas outside the modeling domain from the nominal 3.2 million km^2^ study boundary), is suitable for *B. zonata* (File S8). Suitable habitat was concentrated mainly along the peninsula’s margins rather than its interior (Figure 3). The highest suitability values (≥0.75) were predicted along the southwestern highlands of Saudi Arabia and Yemen, the southeastern coastal plains and mountain ranges of Oman, and the coastal fringes of the United Arab Emirates and the Gulf of Oman. These areas correspond broadly to regions with moderate elevation, lower diurnal temperature range, and substantial cold-season precipitation, the three environmental gradients identified as primary drivers of the species’ distribution (Table 1; Figure 2).

In contrast, most of the Arabian Peninsula interior, including the Rub’ al Khali desert and the central Najd plateau, was predicted as unsuitable or only marginally suitable (suitability < 0.25). This pattern reflects the extreme aridity, large diurnal thermal oscillation, and minimal winter rainfall that characterize these regions. The Red Sea coastal corridor showed a transitional gradient of low to moderate suitability, while isolated high-suitability patches occurred in the Asir and Hejaz mountain ranges along the western escarpment, likely due to cooler and more humid microclimates. Overall, the current distribution indicates that suitable habitat for *B. zonata* is restricted largely to climatically moderated coastal margins and highland zones, whereas the hyper-arid interior functions as an effective barrier to distribution.

### 3.3. Projected Future Habitat Suitability

**Figure 4:**
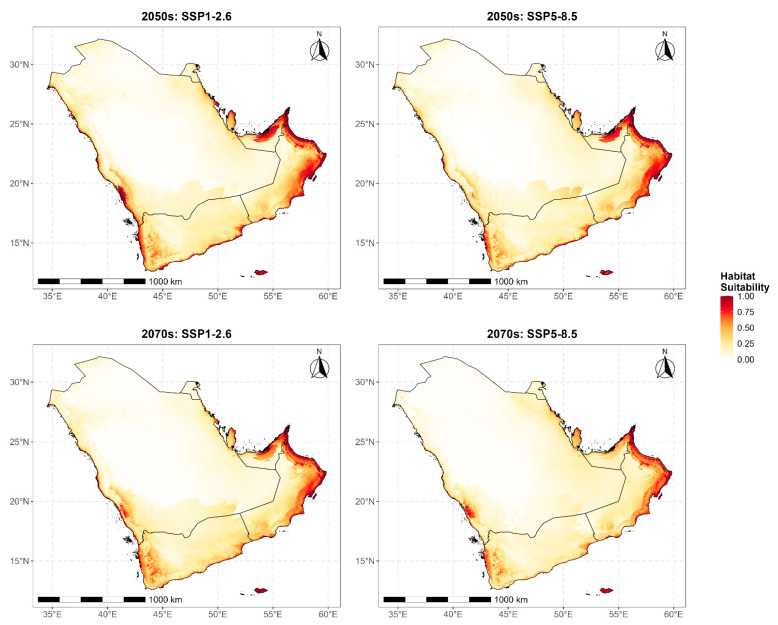
Projected future habitat suitability for *Bactrocera zonata* across the Arabian Peninsula under two Shared Socioeconomic Pathway (SSP1-2.6 and SSP5-8.5) scenarios for the 2050s and 2070s time periods, as predicted by MaxEnt modeling. Habitat suitability values range from 0 (unsuitable, white) to 1 (highly suitable, dark red). Across all scenarios.

Future projections indicated a consistent decline in suitable habitat for *B. zonata* under all climate scenarios and time horizons, with stronger contraction under higher emissions and later periods (Figure 4; File S8). Under SSP1-2.6, suitable area declined from the current 790,714 km^2^ (28.38%) to 732,376 km^2^ (26.28%) by the 2050s and to 722,700 km^2^ (25.94%) by the 2070s. Under SSP5-8.5, the decline was more pronounced, with suitable area reduced to 625,339 km^2^ (22.44%) by the 2050s and 569,265 km^2^ (20.43%) by the 2070s, representing a loss of nearly 28% of the current baseline extent under the most extreme scenario.

**Figure 5:**
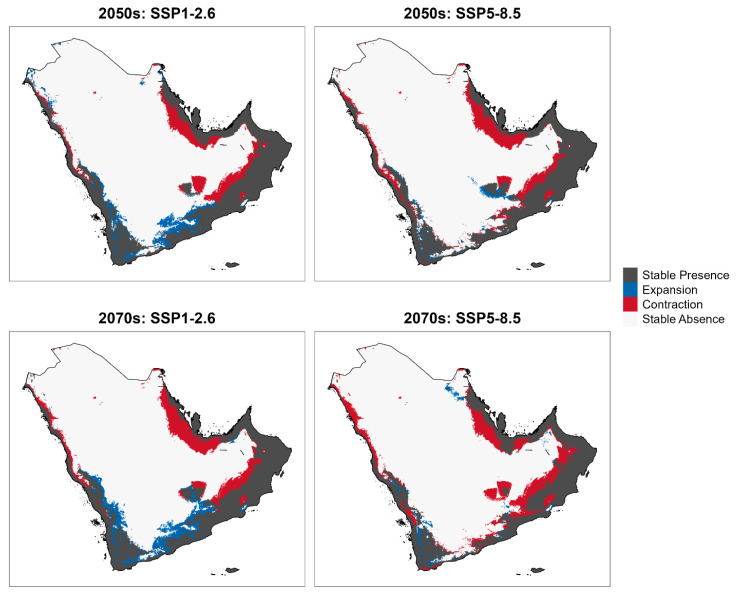
Predicted changes in the climatic suitability of *Bactrocera zonata* across the Arabian Peninsula for the 2050s and 2070s. The multi-panel figure illustrates habitat dynamics under two socio-economic pathways (SSP1-2.6 and SSP5-8.5). Categories denote stable presence (suitable under both current and future conditions), range expansion (newly suitable areas), range contraction (loss of suitability), and stable absence (unsuitable under both conditions).

Range dynamics further showed that contraction dominated all scenario-period combinations (Figure 5). Under SSP1-2.6, contraction accounted for 19.85% and 25.05% of the current suitable area by the 2050s and 2070s, respectively, whereas expansion into newly suitable areas reached 12.48% and 16.44%. Under SSP5-8.5, contraction was substantially greater at 24.67% by the 2050s and 31.80% by the 2070s, while expansion remained limited and nearly unchanged at 3.76% and 3.79%, respectively. These values indicate that stronger warming sharply reduces the scope for compensatory range shifts.

Spatially, contraction was concentrated in the southwestern highlands of Saudi Arabia and Yemen and in parts of the southeastern Omani coast, where areas of high current suitability declined under increasing thermal stress and altered precipitation regimes (Figure 4 and Figure 5). Stable suitable habitat—areas predicted as suitable under both current conditions and future projections—represented the largest single spatial category across all scenarios. Under SSP1-2.6 (2050s), stable suitable habitat covered 633,757 km^2^, which corresponds to approximately 80.2% of current suitable area. Under SSP5-8.5 (2070s), stable suitable habitat declined to 539,267 km^2^ (approximately 68.2% of current suitable area), with the remainder lost to contraction. These values were calculated from the contraction percentages reported in Supplementary File S8. Stable suitable areas were most consistently retained in the eastern Omani mountain ranges and along the Gulf of Oman coastal corridors, which appear to act as climatic refugia under both scenarios. Newly suitable habitat emerged mainly along the northern and northwestern margins of the peninsula under SSP1-2.6, consistent with modest poleward shifts in thermally suitable conditions. Under SSP5-8.5, however, these gains were negligible, suggesting that projected warming exceeds the species’ thermal tolerance across potential colonization zones. Taken together, the projections indicate an overall contraction and spatial reorganization of *B. zonata* habitat across the Arabian Peninsula, with the extent of change determined chiefly by the emission pathway.

## 4. Discussion

The MaxEnt model showed strong discriminatory performance, with mean training and test AUC values of 0.922 and 0.872, respectively, and a mean TSS of 0.538, together indicating a robust ecological signal and a low likelihood of random fit. The modest train-test AUC gap of approximately 0.05 is consistent with regularized MaxEnt behaviour under moderate sample sizes and likely reflects the spatial structure of the occurrence dataset rather than systematic overfitting [13,14]. Selection of the Linear-Quadratic feature class with a regularization multiplier of 0.5, guided by AICc minimization through ENMeval 2.0, yielded a parsimonious model with nine coefficients, sufficient to capture non-linear environmental relationships without overconfiguring response curves to idiosyncratic occurrence clusters [49,51]. Even so, the TSS variability across replicates, ranging from 0.368 to 0.692, cautions against uncritical reliance on single-threshold accuracy metrics in a system where occurrence records are spatially clustered along agricultural corridors, because threshold-dependent statistics are sensitive to both prevalence and the spatial congruence of presences with background samples [56,57]. Interpreting AUC and TSS together across replicates therefore provides a more conservative and defensible basis for confidence in the projections than either metric alone.

Elevation emerged as the dominant predictor of habitat suitability, accounting for 43.4% of model contribution, and this requires mechanistic interpretation within the biogeography of extreme arid systems. It is important to note, however, that in correlative SDMs, elevation functions primarily as a proxy for the underlying climatic gradients it subsumes—particularly temperature lapse rates, reduced thermal maxima, orographic precipitation, and decreased evaporative demand—rather than as a direct physiological driver per se [63]. Because elevation is strongly correlated with temperature and moisture variables even after VIF filtering, its high model contribution may partly reflect co-variation with retained climatic predictors. Formal variance partitioning or partial correlation analysis would be required to disentangle the independent contribution of elevation from collinear climatic gradients, which was beyond the scope of the current analysis. In hyperarid environments such as the Arabian Peninsula, elevation is not merely a physical gradient, but an integrated climatic surrogate that modulates temperature maxima, diurnal thermal amplitude, and moisture availability through orographic precipitation and reduced evaporative demand [63]. The bimodal suitability response along the elevation gradient, with highest suitability at sea level, suppression at 500 to 700 m, and recovery above 1500 m, is ecologically coherent given the contrasting thermal regimes of lowland coastal zones and highland refugia. Coastal lowlands combine high humidity, proximity to fruiting host plants in commercial orchards, and relatively buffered night temperatures, whereas highland zones above 1500 m in the Asir, Oman Highlands, and Yemeni massifs provide reduced thermal maxima and increased cold-season precipitation that extend phenological windows for host fruiting and larval development. The intermediate reduction in suitability at 500 to 700 m corresponds to an ecotonal belt of maximum continental aridity and thermal instability, where interior plateau heating is not offset by sufficient orographic moisture enhancement, consistent with the climatic structure described for the southwestern Saudi Arabian escarpment [27,28]. This bimodal response also parallels the dual-altitude occupancy documented for other polyphagous tephritids in mountainous arid systems and reflects the capacity of *B. zonata* to exploit climatically distinct agricultural zones separated by inhospitable mid-elevation terrain [5,6].

The strong negative relationship between mean diurnal temperature range (Bio 2) and habitat suitability, with conditions exceeding 12 °C diurnal amplitude associated with sharply declining suitability, provides a physiologically grounded explanation for the exclusion of interior desert habitats. Large diurnal oscillations characterize the central Rub’ al Khali and Najd plateau, where daytime surface temperatures can exceed 45 °C and nocturnal radiative cooling drives temperature drops of 20 °C or more [26]. Such extreme thermal variability imposes energetically costly acclimation demands on ectotherms and disrupts developmental synchrony between larval instars and host fruit phenology, a coupling essential for tephritid reproductive success [2]. Experimental thermal acclimation studies on *B. zonata* have shown that the species has moderate heat tolerance relative to other tephritids, but pronounced reductions in fecundity and egg hatching when exposed to temperatures exceeding approximately 34 °C for prolonged periods [2]. This physiological constraint aligns closely with the sharp decline in suitability above a Bio 9 threshold of approximately 28 °C observed in the response curves, reinforcing the mechanistic plausibility of the model and supporting its ecological interpretability rather than treating it as a purely correlative surface. The mean temperature of the driest quarter functioning as a thermal ceiling reflects the compound thermal stress experienced during the summer dry season, when the absence of evapotranspirative cooling in unirrigated landscapes amplifies the effective heat load on insect populations.

Cold-season precipitation (Bio 19) showed a strongly positive sigmoidal relationship with suitability, reaching near-maximum values above 80 mm, underscoring the indirect but critical role of winter moisture in conditioning the distributional limits of this pest. In the Arabian Peninsula, winter precipitation in the southwestern highlands and southeastern Oman supports the phenological expression of fruit crops, particularly mango, guava, and citrus, which constitute the main reproductive substrates for *B. zonata* populations [19,28]. This dependence on cold-season rainfall likely operates primarily through host plant availability rather than direct larval water requirements, because tephritid larvae obtain moisture from fruit pulp rather than ambient precipitation. Such indirect precipitation effects have been documented for other invasive *Bactrocera* species in arid margins, where orchard productivity and seasonal fruit availability act as the proximate determinant of local population growth [6]. The model therefore captures not only direct climatic limitation on insect physiology, but also the compounded constraint imposed by climate on both the pest and its agricultural host system, a distinction often obscured in correlative SDM outputs yet essential for accurate risk assessment in agri-environmental contexts.

The spatial configuration of current habitat suitability reveals a pattern of peripatetic coastal and highland occupancy surrounding a largely inhospitable interior, a distributional architecture that reflects classic climatic filtering in island biogeography applied to desert systems, where suitable habitat patches are effectively isolated by the climatic matrix of the Rub’ al Khali [64,65]. The concentration of high-suitability habitat, defined here as ≥ 0.75, in the Asir–Yemen highland corridor, the Oman Highlands, and the Gulf of Oman coastal fringes identifies these regions as core refugia under present conditions. These areas share orographic moisture enhancement, moderated diurnal range, and intensive cultivation, all of which satisfy the species’ fundamental niche requirements [7]. The Red Sea coastal corridor, which shows transitional moderate suitability, is consistent with historical records of seasonal *B. zonata* activity in Hejaz agricultural areas and reflects a gradient from suitable coastal lowlands to progressively less suitable interior plains. The spatial concordance between modeled current suitability and documented occurrence concentrations in the Eastern Province of Saudi Arabia, the UAE coast, and northern Oman provides empirical validation of the model beyond statistical cross-validation alone.

Future projections under both SSP1-2.6 and SSP5-8.5 indicate a monotonic contraction of suitable habitat through 2070, with net losses of approximately 8.6% and 28.0% of baseline area under low- and high-emission pathways, respectively. These trajectories reveal an asymmetry of theoretical importance: the ratio of contraction to expansion increases markedly from SSP1-2.6, where expansion partially counterbalances 63% of contraction, to SSP5-8.5, where expansion accounts for only 12% of contraction by 2070. It should be noted that these gains and losses occur in geographically distinct areas and do not represent local compensation; rather, they reflect independent spatial processes of suitability gain and loss driven by heterogeneous climate trajectories across the peninsula. This non-linear divergence between scenarios reflects exceedance of critical thermal thresholds in putative range expansion zones, where projected warming under SSP5-8.5 pushes temperatures beyond the species’ upper tolerance limits in northwestern and northern areas where modest colonization was projected under moderate warming [3,66,67]. The concept of climate velocity is potentially relevant here: under SSP5-8.5, it is plausible that the rate of isotherm displacement could exceed the dispersal capacity of *B. zonata* populations in fragmented agricultural landscapes, potentially generating distributional disequilibria in which populations persist in suboptimal conditions or fail to track suitable habitat across climatically hostile corridors [68]. These dispersal-related inferences are necessarily speculative in the absence of quantitative dispersal estimates for *B. zonata* in Arabian Peninsula landscapes, and dedicated dispersal studies would be required to test this hypothesis formally. In the topographically constrained highland systems, it is nonetheless plausible that suitable area becomes compressed into progressively narrower altitudinal bands on mountain slopes, potentially reducing effective patch size for isolated highland populations [69].

The consistent identification of eastern Omani mountain ranges and the Gulf of Oman coastal corridor as stable refugia across all four scenario-period combinations has major implications for invasion management and agricultural biosecurity. These regions represent climatically buffered zones where *B. zonata* is predicted to persist even under severe warming, acting as potential source populations for regional spread into newly adjacent agricultural areas under more moderate seasonal conditions [70]. The Omani highlands, including the Hajar Mountains, have long been recognized as a biodiversity hotspot within the otherwise depauperate peninsula fauna, supporting endemic plant communities and insect assemblages that benefit from orographic interception of Arabian Sea monsoon moisture [71]. The persistence of *B. zonata* suitability in this zone implies continued phytosanitary pressure on the extensive date palm, mango, and tropical fruit cultivations of the Batinah coastal plain, necessitating sustained and adaptive pest management investments regardless of broader regional habitat contraction. Similarly, the projected contraction in the southwestern Saudi–Yemeni highlands, where current suitability is highest, may paradoxically increase pest pressure in residual suitable patches through concentration of populations, a density effect that SDM approaches, which model environmental capacity rather than population dynamics, cannot capture [72].

Comparison with published SDM analyses of related tephritid species situates these findings within broader invasion ecology theory [12] modeled the global distribution of *Bactrocera correcta* under CMIP6 scenarios and documented analogous habitat contraction in hyper-arid Middle Eastern zones under high-emission pathways, while also projecting poleward expansion into temperate agricultural zones, a pattern not observed here because *B. zonata* northward expansion is constrained by the Mediterranean Basin’s existing thermal saturation rather than newly opened climatic space [19] applied integrated MaxEnt approaches to *B. zonata* globally and reported significant shifts in suitability centroids across South and Southeast Asia under SSP8.5 scenarios, broadly consistent with thermal exceedance driving contraction in historically optimal zones. The Arabian Peninsula-specific findings presented here complement those global analyses by resolving fine-scale heterogeneity that continent-scale models necessarily obscure, particularly the dual coastal-highland occupancy architecture and the Rub’ al Khali barrier effect, both of which emerge only at the 1 km resolution used here. It is also noteworthy that previous global models typically relied on coarser climate data (e.g., 2.5–10 arcminute resolution) and, in several cases, did not include elevation as an explicit predictor. These methodological differences may partly explain why mountain refugia in the Omani Highlands and Asir Massif were not prominently identified in global analyses, as their detection appears to depend critically on fine-scale topographic information. Furthermore, ref. [6] similarly documented climate-sensitive range dynamics across the Tephritidae family, with *B. zonata* among the species showing the highest climate sensitivity, a conclusion reinforced by the strong response to the three principal climatic axes identified in the present model. Additional recent studies reinforce key aspects of our findings: He et al. [73] showed that soil factors improve tephritid SDM accuracy, while Liu et al. [74] and Teixeira et al. [75] demonstrated optimized MaxEnt performance and climate-driven range expansion in *Bactrocera diaphora* and *Anastrepha grandis*, respectively. Together, these convergent results support our conclusion that fine-scale environmental heterogeneity and emission pathway are critical for predicting tephritid pest responses to warming.

Several considerations temper inference. Although the dataset was spatially filtered and bias-corrected, it includes 55 georeferenced records, a sample size that is generally sufficient for MaxEnt under appropriate regularization but still limits fine-scale niche resolution and full representation of the regional environmental space occupied by *B. zonata* [59]. While MaxEnt has demonstrated reasonable performance with datasets of this size under suitable regularization, marginal response curves derived from small occurrence datasets should be interpreted with caution, as they may inadequately characterize species’ true environmental tolerances and may be sensitive to the particular subset of occurrences used in model calibration. Spatial gaps in Yemen and inland Saudi Arabia likely reflect uneven survey effort rather than true absence, so suitability there may be conservatively estimated despite the bias file. The model also assumes that observed occurrences approximate realized climatic tolerances; incomplete colonization could therefore yield conservative niche estimates [9]. In addition, the absence of biotic interactions means the projections represent potential climatic suitability rather than realized distributions shaped by community-level processes, including competition and parasitoid pressure, particularly in climatically buffered highland environments [3,7]. An additional implicit assumption is that host plant availability remains constant across the projection period; however, climate-driven shifts in the cultivation range or phenology of key hosts (mango, guava, citrus, and peach) could alter the realized distribution of *B. zonata* independently of direct climatic constraints, a dynamic that correlative SDMs cannot accommodate without explicit host-plant modeling. Although host plant distribution and anthropogenic dispersal are not quantitatively incorporated here, their potential influence is acknowledged; future work should integrate spatially explicit host data and dispersal networks. Likewise, near-term projections (e.g., 2030s) were not produced because the chosen GCM scenarios standardly target 2050 and 2070, but such shorter-horizon maps would enhance immediate management applicability and are recommended for follow-up studies.

The use of 30 arc-second, approximately 1 km^2^, climate surfaces represents a substantial improvement over earlier GBIF-based global analyses, but it still smooths the topographic heterogeneity of steep mountain systems where fine-scale microclimatic stability, from meters to tens of meters, may sustain populations in climatically hostile grid cells [76]. Downscaled microclimatic modeling integrated with mechanistic thermal tolerance data would substantially improve predictive precision for highland refugia, where divergence between macroclimatic grids and operative temperatures experienced by insects is greatest. For example, on steep south-facing versus north-facing slopes in the Hajar Mountains, surface temperatures can differ by 5–10 °C within tens of meters, creating microclimatic refugia that coarse-resolution grids cannot resolve. Future work would benefit from incorporating fine-resolution climate and topographic datasets, such as 90 m or 30 m digital elevation models combined with statistically downscaled climate surfaces at sub-kilometer resolution, to better characterize the refugial capacity of mountain systems. Uncertainty in future climate projections, while partly addressed through the five-GCM ensemble approach, remains an irreducible source of variation, particularly for precipitation over the Arabian Peninsula, where inter-model spread in CMIP6 rainfall outputs is disproportionately large relative to temperature [22]. The multi-model mean approach adopted here reduces but does not eliminate this uncertainty, and probability distributions of suitability change would more rigorously characterize the envelope of plausible outcomes for management planning.

Collectively, these findings carry direct implications for agricultural biosecurity governance across the Arabian Peninsula. The persistence of suitable habitat in climatically buffered highland and coastal zones under all scenarios suggests that *B. zonata* will remain a sustained phytosanitary threat irrespective of emission pathway, and that management planning premised on climate-driven pest decline would be misguided. The asymmetry between SSP pathways, in which strong emissions mitigation (SSP1-2.6) retains substantially more suitable habitat while strong warming (SSP5-8.5) compresses the distribution into refugial patches with uncertain population dynamics, illustrates the non-linear relationship between global climate policy and regional pest risk. National plant protection organizations across Saudi Arabia, Oman, and the UAE should incorporate projected suitability maps into adaptive surveillance network design, prioritizing monitoring in climatically stable coastal and highland zones where populations are predicted to persist, while maintaining border biosecurity for potential reintroduction from adjacent invasion corridors in North Africa and South Asia [5,20]. Integration of species distribution modeling outputs with population dynamic models and economic impact assessments is therefore a logical and necessary next step for translating the spatial predictions generated here into actionable risk management frameworks for the region’s economically vital horticultural sector.

## 5. Conclusions

Regional warming will not homogenously contract *B. zonata* populations across the Arabian Peninsula but will instead force a fundamental spatial reorganization of climatically viable habitat, compressing persistence into orographically buffered highland and coastal refugia while eliminating suitability across thermally saturated lowland and interior zones. The nonlinear divergence between SSP1-2.6 and SSP5-8.5 trajectories reveals a threshold-structured response to anthropogenic forcing, in which range expansion only partially counterbalances contraction under moderate warming, and becomes negligible under high emissions. Critically, refugial persistence in the Hajar and Asir massifs guarantees continued propagule pressure on surrounding agricultural systems regardless of emission pathway, rendering climate-driven optimism about pest attrition biologically unjustified. Adaptive biosecurity governance must therefore be anchored in spatial predictions of refugial stability rather than aggregate suitability trends.

## Figures and Tables

**Table 1:** Percentage contributions and permutation importance values of selected environmental predictor variables included in the MaxEnt species distribution models for *Bactrocera zonata*, estimated across ten replicate model runs.

Variables	Description	% Contribution	Permutation Importance
Elev	Altitude in meters	43.4	29.8
Bio 9	Mean temperature of driest quarter	18.8	24.2
Bio 2	Mean diurnal range	18.7	20.4
Bio 19	Precipitation of coldest quarter	16	19.1
Bio 8	Mean temperature of wettest quarter	1.9	4.1
Bio 15	Precipitation seasonality (coefficient of variation)	0.8	1.2
Bio 3	Isothermality	0.3	1.1

## Data Availability

Data supporting this study are included within the article and/or its Supplementary Materials.

## Supplementary Materials

The following supporting information can be downloaded at: https://www.mdpi.com/article/10.3390/biology15100814/s1, File S1: Occurrence records of *Bactrocera zonata* in the Arabian Peninsula; File S2: R scripts for spatial thinning, bioclimatic variable calculation, VIF analysis, layer preprocessing, and ENMeval model tuning; File S3: List of environmental variables used in distribution modeling; File S4: Variance Inflation Factor (VIF) analysis table showing variable selection; File S5: Details of the five General Circulation Models (GCMs) used for future projections; File S6: ENMeval tuning results (feature classes, regularization multipliers, AICc); File S7: Replicate-level MaxEnt performance metrics (AUC, TSS); File S8: Projected current and future suitable habitat extent (km^2^ and %) under SSP1-2.6 and SSP5-8.5 for 2050 and 2070; Figure S1: ROC curve of MaxEnt model performance; Figure S2: Jackknife test of variable importance.

## Abbreviations

The following abbreviations are used in this manuscript:

AICc	Corrected Akaike Information Criterion
ASCII	American Standard Code for Information Interchange
ASTER	Advanced Spaceborne Thermal Emission and Reflection Radiometer
ASTWBD	ASTER Global Water Body Dataset
AUC	Area Under the Receiver Operating Characteristic Curve
Bio 2	Mean Diurnal Temperature Range
Bio 3	Isothermality
Bio 8	Mean Temperature of the Wettest Quarter
Bio 9	Mean Temperature of the Driest Quarter
Bio 15	Precipitation Seasonality
Bio 19	Precipitation of the Coldest Quarter
BOLD	Barcode of Life Data System
CMIP6	Coupled Model Intercomparison Project Phase 6
Elev	Elevation
ENMeval	Ecological Niche Model Evaluation
EPPO	European and Mediterranean Plant Protection Organization
FC	Feature Class
GBIF	Global Biodiversity Information Facility
GCM	General Circulation Model
GDEM	Global Digital Elevation Model
GEE	Google Earth Engine
GIS	Geographic Information System
ISIMIP3b	Inter-Sectoral Impact Model Intercomparison Project Phase 3b
L	Linear (feature class)
LQ	Linear-Quadratic (feature class)
LQH	Linear-Quadratic-Hinge (feature class)
LQHP	Linear-Quadratic-Hinge-Product (feature class)
H	Hinge (feature class)
MaxEnt	Maximum Entropy (modeling framework)
MENA	Middle East and North Africa
RM	Regularization Multiplier
ROC	Receiver Operating Characteristic
ScenarioMIP	Scenario Model Intercomparison Project
SDM	Species Distribution Model
SDMtoolbox	Species Distribution Model Toolbox
SSP	Shared Socioeconomic Pathway
SSP1-2.6	Shared Socioeconomic Pathway 1—radiative forcing target of 2.6 W m^−2^
SSP5-8.5	Shared Socioeconomic Pathway 5—radiative forcing target of 8.5 W m^−2^
TSS	True Skill Statistic
UAE	United Arab Emirates
UN ESCWA	United Nations Economic and Social Commission for Western Asia
VIF	Variance Inflation Factor
