# Supplementary material for: Climatic Niche Contraction and Refugial Persistence of an Invasive Tephritid Pest Across the Arabian Peninsula Under Contrasting Emission Scenarios
Source: Biology (Basel). 2026 May 21;15(10):814. doi: 10.3390/biology15100814 (PMC13203219; doi:10.3390/biology15100814)
Supplement: Supplementary file 1 [file biology-15-00814-s001.zip › File S3.docx]

**S3 File.** List of variables used in the distribution modeling process of *Bactrocera zonata*.

| Variable | Description | Unit | Source |
| --- | --- | --- | --- |
| Bio1 | Annual mean temperature | °C | TerraClimate ^a^ |
| Bio2 | Mean diurnal range (max temp − min temp) (monthly average) | °C |  |
| Bio3 | Isothermality (Bio1/Bio7) × 100 | % |  |
| Bio4 | Temperature seasonality (coefficient of variation) | % |  |
| Bio5 | Max temperature of warmest period | °C |  |
| Bio6 | Min temperature of the coldest period | °C |  |
| Bio7 | Temperature annual range | °C |  |
| Bio8 | Mean temperature of wettest quarter | °C |  |
| Bio9 | Mean temperature of driest quarter | °C |  |
| Bio10 | Mean temperature of warmest quarter | °C |  |
| Bio11 | Mean temperature of coldest quarter | °C |  |
| Bio12 | Annual precipitation | mm |  |
| Bio13 | Precipitation of wettest period | mm |  |
| Bio14 | Precipitation of driest period | mm |  |
| Bio15 | Precipitation seasonality (coefficient of variation) | % |  |
| Bio16 | Precipitation of wettest quarter | mm |  |
| Bio17 | Precipitation of driest quarter | mm |  |
| Bio18 | Precipitation of warmest quarter | mm |  |
| Bio19 | Precipitation of coldest quarter | mm |  |
| Alt | Altitude in meters | m | WorldClim ^b^ |

^a^ [TerraClimate - Climatology Lab](https://www.climatologylab.org/terraclimate.html)

^b^ <http://www.worldclim.org/bioclim>
