# Supplementary material for: Climatic Niche Contraction and Refugial Persistence of an Invasive Tephritid Pest Across the Arabian Peninsula Under Contrasting Emission Scenarios
Source: Biology (Basel). 2026 May 21;15(10):814. doi: 10.3390/biology15100814 (PMC13203219; doi:10.3390/biology15100814)
Supplement: Supplementary file 1 [file biology-15-00814-s001.zip › File S2.docx]

Spatial Filtering of Occurrence Data Using spThin

# Spatial thinning of Bactrocera zonata occurrence data (5 km)

# Reduces spatial autocorrelation by removing points within 5 km of each other

if (!require("spThin")) install.packages("spThin", repos = "https://cran.r-project.org")

library(spThin)

# Read occurrence data

occ <- read.csv("c:/Users/solim/Desktop/AP/Bactrocera_zonata/Occurrence/occ.csv",

                 stringsAsFactors = FALSE)

cat("Original number of records:", nrow(occ), "\n")

# Run spatial thinning with 5 km minimum distance

set.seed(42)

thinned <- thin(

  loc.data = occ,

  lat.col = "Latitude",

  long.col = "Longitude",

  spec.col = "Country",

  thin.par = 5,        # 5 km minimum distance

  reps = 100,          # number of repetitions to find optimal set

  locs.thinned.list.return = TRUE,

  write.files = FALSE,

  write.log.file = FALSE

)

# Select the replicate that retained the most records

max_idx <- which.max(sapply(thinned, nrow))

best_thinned <- thinned[[max_idx]]

cat("Records after thinning (5 km):", nrow(best_thinned), "\n")

# Merge back with original data to retain all columns

occ_thinned <- merge(best_thinned, occ,

                     by.x = c("Latitude", "Longitude"),

                     by.y = c("Latitude", "Longitude"),

                     all.x = TRUE)

# Remove any duplicates from merging

occ_thinned <- occ_thinned[!duplicated(occ_thinned[, c("Latitude", "Longitude")]), ]

# Reorder columns

occ_thinned <- occ_thinned[, c("Country", "Locality", "Latitude", "Longitude", "Source", "Year")]

# Sort by Country, then Year

occ_thinned <- occ_thinned[order(occ_thinned$Country, occ_thinned$Year), ]

cat("\nThinned records by country:\n")

print(table(occ_thinned$Country))

# Save thinned data

write.csv(occ_thinned,

          "c:/Users/solim/Desktop/AP/Bactrocera_zonata/Occurrence/occ_thinned.csv",

          row.names = FALSE)

cat("\nThinned occurrence data saved to: occ_thinned.csv\n")

cat("Records removed:", nrow(occ) - nrow(occ_thinned), "\n")

Computing of Bioclimatic Variables from Long-Term Climate Data

################################################################################

# Script: Calculate Bioclimatic Variables

# Purpose: Calculate BIO variables using terra for memory efficiency

# Study: Bactrocera zonata distribution modeling in the Arabian Peninsula

################################################################################

# Clear environment

rm(list = ls())

gc()

# Install and load required packages

packages <- c("terra")

# Function to install missing packages

install_if_missing <- function(package) {

  if (!require(package, character.only = TRUE)) {

    options(repos = c(CRAN = "https://cloud.r-project.org"))

    install.packages(package, dependencies = TRUE)

    library(package, character.only = TRUE)

  }

}

# Install and load packages

for (pkg in packages) {

  install_if_missing(pkg)

}

cat("All required packages loaded successfully!\n\n")

################################################################################

# SECTION 1: SET PATHS AND PARAMETERS

################################################################################

# Define input paths (Windows format)

path_tmax <- "C:/Users/solim/Desktop/AP/Bactrocera_zonata/max"

path_tmin <- "C:/Users/solim/Desktop/AP/Bactrocera_zonata/min"

path_prec <- "C:/Users/solim/Desktop/AP/Bactrocera_zonata/prec"

# Define output path

output_path <- "C:/Users/solim/Desktop/AP/Bactrocera_zonata/bioclim_output"

# Create output directory if it doesn't exist

if (!dir.exists(output_path)) {

  dir.create(output_path, recursive = TRUE)

}

cat("Output directory:", output_path, "\n")

################################################################################

# SECTION 2: LOAD CLIMATE DATA

################################################################################

cat("\nSTEP 1: Loading climate data with terra...\n")

tmax_files <- sort(list.files(path_tmax, pattern = "\\.tif$|\\.asc$", full.names = TRUE))

tmin_files <- sort(list.files(path_tmin, pattern = "\\.tif$|\\.asc$", full.names = TRUE))

prec_files <- sort(list.files(path_prec, pattern = "\\.tif$|\\.asc$", full.names = TRUE))

if (length(tmax_files) != 12 || length(tmin_files) != 12 || length(prec_files) != 12) {

  stop("ERROR: Expected 12 monthly files for each variable.")

}

tmax <- terra::rast(tmax_files)

tmin <- terra::rast(tmin_files)

prec <- terra::rast(prec_files)

cat("Climate data loaded successfully!\n")

################################################################################

# SECTION 3: CALCULATE ALL 19 BIO VARIABLES

################################################################################

cat("\nSTEP 2: Calculating all 19 bioclimatic variables (BIO1 to BIO19)...\n")

# --- 1. Basic Temperature Variables ---

cat("  - Calculating BIO1 to BIO7...\n")

tmean <- (tmax + tmin) / 2

names(tmean) <- month.abb

# BIO 1: Annual Mean Temperature

bio1 <- terra::app(tmean, fun = "mean")

names(bio1) <- "BIO1"

# BIO 2: Mean Diurnal Range (Mean of monthly (max temp - min temp))

bio2 <- terra::app(tmax - tmin, fun = "mean")

names(bio2) <- "BIO2"

# BIO 5: Max Temperature of Warmest Month

bio5 <- terra::app(tmax, fun = "max")

names(bio5) <- "BIO5"

# BIO 6: Min Temperature of Coldest Month

bio6 <- terra::app(tmin, fun = "min")

names(bio6) <- "BIO6"

# BIO 7: Temperature Annual Range (BIO5-BIO6)

bio7 <- bio5 - bio6

names(bio7) <- "BIO7"

# BIO 3: Isothermality (BIO2/BIO7) * 100

bio3 <- (bio2 / bio7) * 100

names(bio3) <- "BIO3"

# BIO 4: Temperature Seasonality (standard deviation * 100)

# WorldClim definition uses SD of monthly means

bio4 <- terra::app(tmean, fun = "sd") * 100

names(bio4) <- "BIO4"

# --- 2. Basic Precipitation Variables ---

cat("  - Calculating BIO12 to BIO15...\n")

# BIO 12: Annual Precipitation

bio12 <- terra::app(prec, fun = "sum")

names(bio12) <- "BIO12"

# BIO 13: Precipitation of Wettest Month

bio13 <- terra::app(prec, fun = "max")

names(bio13) <- "BIO13"

# BIO 14: Precipitation of Driest Month

bio14 <- terra::app(prec, fun = "min")

names(bio14) <- "BIO14"

# BIO 15: Precipitation Seasonality (Coefficient of Variation)

# CV = (SD / Mean) * 100. Adding a small epsilon to avoid division by zero.

bio15 <- terra::lapp(prec, fun = function(x) {

  m <- rowMeans(x, na.rm = TRUE)

  s <- apply(x, 1, sd, na.rm = TRUE)

  res <- (s / (m + 0.000001)) * 100

  return(res)

})

names(bio15) <- "BIO15"

# --- 3. Quarter-based Variables (BIO8-11, BIO16-19) ---

cat("  - Calculating Quarter-based variables (BIO8-11, BIO16-19)...\n")

# Handle circularity for quarters (e.g., Dec-Jan-Feb)

tmean_ext <- c(tmean, tmean[[1:2]])

prec_ext <- c(prec, prec[[1:2]])

q_tmean_list <- list()

q_prec_list <- list()

for (i in 1:12) {

  q_tmean_list[[i]] <- terra::mean(tmean_ext[[i:(i + 2)]])

  q_prec_list[[i]] <- terra::sum(prec_ext[[i:(i + 2)]])

}

q_tmean_stack <- terra::rast(q_tmean_list)

q_prec_stack <- terra::rast(q_prec_list)

# Indices for Wettest, Driest, Warmest, Coldest quarters

wet_q_idx <- terra::app(q_prec_stack, fun = "which.max")

dry_q_idx <- terra::app(q_prec_stack, fun = "which.min")

warm_q_idx <- terra::app(q_tmean_stack, fun = "which.max")

cold_q_idx <- terra::app(q_tmean_stack, fun = "which.min")

# Helper function to extract values from a stack based on an index layer

pick_val <- function(stack, idx) {

  terra::lapp(c(stack, idx), fun = function(...) {

    dots <- list(...)

    s <- do.call(cbind, dots[1:12])

    i <- dots[[13]]

    res <- rep(NA, length(i))

    valid <- !is.na(i)

    if (any(valid)) {

      res[valid] <- s[cbind(which(valid), i[valid])]

    }

    return(res)

  })

}

# Temperature of quarters

bio8  <- pick_val(q_tmean_stack, wet_q_idx)  ; names(bio8) <- "BIO8"  # Mean Temp of Wettest Quarter

bio9  <- pick_val(q_tmean_stack, dry_q_idx)  ; names(bio9) <- "BIO9"  # Mean Temp of Driest Quarter

bio10 <- pick_val(q_tmean_stack, warm_q_idx) ; names(bio10) <- "BIO10" # Mean Temp of Warmest Quarter

bio11 <- pick_val(q_tmean_stack, cold_q_idx) ; names(bio11) <- "BIO11" # Mean Temp of Coldest Quarter

# Precipitation of quarters

bio16 <- pick_val(q_prec_stack, wet_q_idx)   ; names(bio16) <- "BIO16" # Precip of Wettest Quarter

bio17 <- pick_val(q_prec_stack, dry_q_idx)   ; names(bio17) <- "BIO17" # Precip of Driest Quarter

bio18 <- pick_val(q_prec_stack, warm_q_idx)  ; names(bio18) <- "BIO18" # Precip of Warmest Quarter

bio19 <- pick_val(q_prec_stack, cold_q_idx)  ; names(bio19) <- "BIO19" # Precip of Coldest Quarter

# Combine all layers

bioclim_terra <- c(

  bio1, bio2, bio3, bio4, bio5, bio6, bio7, bio8, bio9, bio10,

  bio11, bio12, bio13, bio14, bio15, bio16, bio17, bio18, bio19

)

bioclim_names <- c(

  "BIO01_Annual_Mean_Temperature",

  "BIO02_Mean_Diurnal_Range",

  "BIO03_Isothermality",

  "BIO04_Temperature_Seasonality",

  "BIO05_Max_Temperature_Warmest_Month",

  "BIO06_Min_Temperature_Coldest_Month",

  "BIO07_Temperature_Annual_Range",

  "BIO08_Mean_Temperature_Wettest_Quarter",

  "BIO09_Mean_Temperature_Driest_Quarter",

  "BIO10_Mean_Temperature_Warmest_Quarter",

  "BIO11_Mean_Temperature_Coldest_Quarter",

  "BIO12_Annual_Precipitation",

  "BIO13_Precipitation_Wettest_Month",

  "BIO14_Precipitation_Driest_Month",

  "BIO15_Precipitation_Seasonality",

  "BIO16_Precipitation_Wettest_Quarter",

  "BIO17_Precipitation_Driest_Quarter",

  "BIO18_Precipitation_Warmest_Quarter",

  "BIO19_Precipitation_Coldest_Quarter"

)

cat("Calculations completed for all 19 variables!\n")

################################################################################

# SECTION 4: SAVE RESULTS

################################################################################

cat("\nSTEP 3: Saving results...\n")

for (i in 1:terra::nlyr(bioclim_terra)) {

  fname <- file.path(output_path, paste0(bioclim_names[i], ".tif"))

  terra::writeRaster(bioclim_terra[[i]], filename = fname, overwrite = TRUE, gdal = c("COMPRESS=LZW"))

  cat("  Saved:", bioclim_names[i], "\n")

}

# Save as a multi-band TIFF as well

terra::writeRaster(bioclim_terra,

  filename = file.path(output_path, "All_Bioclim_Variables_1_to_19.tif"),

  overwrite = TRUE,

  gdal = c("COMPRESS=LZW")

)

cat("\nSTEP 4: Generating summary statistics...\n")

summary_stats <- data.frame(

  Variable = bioclim_names,

  Min = as.vector(terra::global(bioclim_terra, "min", na.rm = TRUE)[, 1]),

  Max = as.vector(terra::global(bioclim_terra, "max", na.rm = TRUE)[, 1]),

  Mean = as.vector(terra::global(bioclim_terra, "mean", na.rm = TRUE)[, 1]),

  SD = as.vector(terra::global(bioclim_terra, "sd", na.rm = TRUE)[, 1])

)

print(summary_stats)

write.csv(summary_stats, file = file.path(output_path, "Bioclim_Full_Statistics.csv"), row.names = FALSE)

cat("\nAll processes completed successfully at:", as.character(Sys.time()), "\n")

Multicollinearity Assessment Using Variance Inflation Factor (VIF)

# =============================================================================

# VIF Analysis for Bactrocera zonata SDM

# Paper: "Climate-driven distribution modeling of Peach Fruit Fly

#         (Bactrocera zonata) in the Arabian Peninsula: Current patterns

#         and future projections"

# =============================================================================

# --- Load required packages ---

required_packages <- c("terra", "usdm", "dplyr", "flextable", "officer")

for (pkg in required_packages) {

  if (!requireNamespace(pkg, quietly = TRUE)) install.packages(pkg)

  library(pkg, character.only = TRUE)

}

# --- 1. Set paths ---

raster_dir  <- "C:/Users/solim/Desktop/AP/current"

occ_file    <- "C:/Users/solim/Desktop/AP/Bactrocera_zonata/occ.csv"

output_dir  <- "C:/Users/solim/Desktop/AP/Bactrocera_zonata/Results"

if (!dir.exists(output_dir)) dir.create(output_dir, recursive = TRUE)

# --- 2. Load rasters as a SpatRaster stack ---

tif_files <- list.files(raster_dir, pattern = "\\.tif$", full.names = TRUE)

cat("Loading", length(tif_files), "raster layers...\n")

env_stack <- rast(tif_files)

# Ensure layer names match filenames exactly (e.g., "Bio1", "Bio2")

names(env_stack) <- tools::file_path_sans_ext(basename(tif_files))

print(names(env_stack))

# --- 3. Load occurrence points ---

occ <- read.csv(occ_file)

cat("Occurrence records loaded:", nrow(occ), "\n")

# --- 4. Extract raster values at occurrence points ---

occ_coords <- vect(occ, geom = c("Longitude", "Latitude"), crs = "EPSG:4326")

env_values <- terra::extract(env_stack, occ_coords)

env_values <- env_values[, -1]  # remove ID column

env_values <- na.omit(env_values)  # remove rows with NA

cat("Extracted values for", nrow(env_values), "points (after NA removal)\n")

# --- 5. VIF analysis BEFORE variable removal (all 20 variables) ---

cat("\n=== VIF BEFORE variable removal ===\n")

vif_before <- vif(env_values)

print(vif_before)

# --- 6. Stepwise VIF exclusion with threshold = 5 ---

cat("\n=== Stepwise VIF exclusion (threshold = 5) ===\n")

vif_step <- vifstep(env_values, th = 5)

print(vif_step)

# --- 7. VIF AFTER variable removal (retained variables only) ---

retained_vars  <- vif_step@results$Variables

excluded_vars  <- setdiff(names(env_values), retained_vars)

cat("\nRetained variables:", paste(retained_vars, collapse = ", "), "\n")

cat("Excluded variables:", paste(excluded_vars, collapse = ", "), "\n")

env_retained <- env_values[, retained_vars, drop = FALSE]

vif_after <- vif(env_retained)

cat("\n=== VIF AFTER variable removal ===\n")

print(vif_after)

# --- 8. Build publication-ready table ---

# Full descriptive names for bioclimatic variables

var_descriptions <- c(

  Bio1  = "Annual Mean Temperature",

  Bio2  = "Mean Diurnal Range",

  Bio3  = "Isothermality",

  Bio4  = "Temperature Seasonality",

  Bio5  = "Max Temperature of Warmest Month",

  Bio6  = "Min Temperature of Coldest Month",

  Bio7  = "Temperature Annual Range",

  Bio8  = "Mean Temperature of Wettest Quarter",

  Bio9  = "Mean Temperature of Driest Quarter",

  Bio10 = "Mean Temperature of Warmest Quarter",

  Bio11 = "Mean Temperature of Coldest Quarter",

  Bio12 = "Annual Precipitation",

  Bio13 = "Precipitation of Wettest Month",

  Bio14 = "Precipitation of Driest Month",

  Bio15 = "Precipitation Seasonality",

  Bio16 = "Precipitation of Wettest Quarter",

  Bio17 = "Precipitation of Driest Quarter",

  Bio18 = "Precipitation of Warmest Quarter",

  Bio19 = "Precipitation of Coldest Quarter",

  Elev  = "Elevation"

)

# Create the table data frame

before_df <- data.frame(

  Variable    = as.character(vif_before$Variables),

  VIF_Before  = round(vif_before$VIF, 2),

  stringsAsFactors = FALSE

)

after_df <- data.frame(

  Variable   = as.character(vif_after$Variables),

  VIF_After  = round(vif_after$VIF, 2),

  stringsAsFactors = FALSE

)

# Merge

table_df <- merge(before_df, after_df, by = "Variable", all.x = TRUE)

# Add description column

table_df$Description <- var_descriptions[table_df$Variable]

# Mark excluded variables

table_df$Status <- ifelse(is.na(table_df$VIF_After), "Excluded", "Retained")

# Replace NA VIF_After with "—"

table_df$VIF_After_display <- ifelse(is.na(table_df$VIF_After), "\u2014",

                                     as.character(table_df$VIF_After))

# Order by Variable (natural numeric sort)

table_df$sort_key <- as.numeric(gsub("[^0-9]", "", table_df$Variable))

table_df$sort_key[is.na(table_df$sort_key)] <- 99  # Elev last

table_df <- table_df[order(table_df$sort_key), ]

rownames(table_df) <- NULL

# Final table for publication

pub_table <- data.frame(

  Variable         = table_df$Variable,

  Description      = table_df$Description,

  `VIF (Before)`   = table_df$VIF_Before,

  `VIF (After)`    = table_df$VIF_After_display,

  Status           = table_df$Status,

  check.names      = FALSE,

  stringsAsFactors  = FALSE

)

# --- 9. Save as CSV ---

csv_path <- file.path(output_dir, "VIF_Analysis_Table.csv")

write.csv(pub_table, csv_path, row.names = FALSE)

cat("\nCSV table saved to:", csv_path, "\n")

# --- 10. Create a professional Word table using flextable ---

ft <- flextable(pub_table)

ft <- set_header_labels(ft,

  Variable       = "Variable",

  Description    = "Description",

  `VIF (Before)` = "VIF (Before Selection)",

  `VIF (After)`  = "VIF (After Selection)",

  Status         = "Status"

)

# Add a spanning header

ft <- add_header_row(ft,

  values = c("Table 1. Variance Inflation Factor (VIF) analysis of environmental variables used in the distribution modeling of Bactrocera zonata in the Arabian Peninsula. Variables with VIF > 5 were iteratively removed."),

  colwidths = 5

)

# Style the table

ft <- theme_booktabs(ft)

ft <- fontsize(ft, size = 10, part = "all")

ft <- fontsize(ft, size = 9, i = 1, part = "header")

ft <- font(ft, fontname = "Times New Roman", part = "all")

ft <- align(ft, j = c(1, 4, 5), align = "center", part = "all")

ft <- align(ft, j = 2, align = "left", part = "body")

ft <- align(ft, j = 3, align = "center", part = "body")

ft <- bold(ft, part = "header")

ft <- italic(ft, i = 1, part = "header")  # table caption italic

# Highlight excluded rows

excluded_rows <- which(pub_table$Status == "Excluded")

if (length(excluded_rows) > 0) {

  ft <- color(ft, i = excluded_rows, color = "#B22222", part = "body")

}

# Highlight retained rows

retained_rows <- which(pub_table$Status == "Retained")

if (length(retained_rows) > 0) {

  ft <- bg(ft, i = retained_rows, bg = "#F0FFF0", part = "body")

}

# Column widths

ft <- width(ft, j = 1, width = 0.8)

ft <- width(ft, j = 2, width = 2.8)

ft <- width(ft, j = 3, width = 1.3)

ft <- width(ft, j = 4, width = 1.3)

ft <- width(ft, j = 5, width = 0.8)

# Add footer

ft <- add_footer_lines(ft,

  values = "VIF threshold = 5. Variables with VIF > 5 were iteratively excluded using the vifstep function from the usdm package in R."

)

ft <- fontsize(ft, size = 8, part = "footer")

ft <- italic(ft, part = "footer")

# Save as Word document

docx_path <- file.path(output_dir, "VIF_Analysis_Table.docx")

doc <- read_docx()

doc <- body_add_flextable(doc, ft)

print(doc, target = docx_path)

cat("Word table saved to:", docx_path, "\n")

# --- 11. Save retained variable names for downstream use ---

retained_path <- file.path(output_dir, "retained_variables.txt")

writeLines(retained_vars, retained_path)

cat("Retained variable names saved to:", retained_path, "\n")

# --- 12. Summary ---

cat("\n========================================\n")

cat("VIF ANALYSIS SUMMARY\n")

cat("========================================\n")

cat("Total variables:    ", nrow(pub_table), "\n")

cat("Retained variables: ", sum(pub_table$Status == "Retained"), "\n")

cat("Excluded variables: ", sum(pub_table$Status == "Excluded"), "\n")

cat("VIF threshold:       5\n")

cat("========================================\n")

cat("Excluded: ", paste(excluded_vars, collapse = ", "), "\n")

cat("Retained: ", paste(retained_vars, collapse = ", "), "\n")

cat("========================================\n")

Preprocessing of Environmental Layers for Modeling

# Install and load necessary packages

if (!require("terra")) {

    install.packages("terra", repos = "https://cloud.r-project.org")

    library(terra)

}

# Define paths

bioclim_path <- "C:/Users/solim/Desktop/AP/Bactrocera_zonata/bioclim"

current_path <- "C:/Users/solim/Desktop/AP/Bactrocera_zonata/current"

output_path <- "C:/Users/solim/Desktop/AP/Bactrocera_zonata/bioclim_clipped"

# Create output directory if it doesn't exist

if (!dir.exists(output_path)) {

    dir.create(output_path, recursive = TRUE)

    cat("Created output directory:", output_path, "\n")

}

# List files

bioclim_files <- list.files(bioclim_path, pattern = "\\.tif$", full.names = TRUE, ignore.case = TRUE)

# Exclude .ovr files just in case regex picks them up, though $ shouldn't.

bioclim_files <- bioclim_files[!grepl("\\.ovr", bioclim_files)]

current_files <- list.files(current_path, pattern = "\\.tif$", full.names = TRUE, ignore.case = TRUE)

if (length(current_files) == 0) {

    stop("No reference .tif files found in ", current_path)

}

# Use the first file in 'current' as the reference for extent and resolution

reference_raster <- rast(current_files[1])

cat("Using reference raster:", current_files[1], "\n")

cat("Reference Extent:\n")

print(ext(reference_raster))

cat("Reference Resolution:", res(reference_raster), "\n")

cat("Reference CRS:", crs(reference_raster, proj = TRUE), "\n")

# Process each bioclim file

for (file in bioclim_files) {

    fname <- basename(file)

    cat("Processing:", fname, "\n")

    r <- rast(file)

    # Resample to match resolution and extent of reference

    # 'near' is usually good for categorical, 'bilinear' for continuous.

    # Bioclim variables are continuous.

    # Check if projection matches, if not, project first.

    if (crs(r) != crs(reference_raster)) {

        cat("  Reprojecting to match CRS...\n")

        r <- project(r, reference_raster)

    }

    # Resample and crop

    # resample() aligns the new raster to the origin and resolution of the target

    r_resampled <- resample(r, reference_raster, method = "bilinear")

    # Mask to the same valid areas (if current rasters have NAs outside the study area)

    r_masked <- mask(r_resampled, reference_raster)

    # Save the output

    out_file <- file.path(output_path, fname)

    writeRaster(r_masked, out_file, overwrite = TRUE)

    cat("  Saved to:", out_file, "\n")

}

cat("Processing complete.\n")

library(terra)

# Define directories

input_dir <- "c:/Users/solim/Desktop/AP/Bactrocera_zonata/bioclim_clipped"

output_dir <- "c:/Users/solim/Desktop/AP/Bactrocera_zonata/bioclim_asc"

# Create output directory if it doesn't exist

if (!dir.exists(output_dir)) {

    dir.create(output_dir, recursive = TRUE)

    cat("Created output directory:", output_dir, "\n")

} else {

    cat("Output directory already exists:", output_dir, "\n")

}

# List all tif files

tif_files <- list.files(input_dir, pattern = "\\.tif$", full.names = TRUE)

if (length(tif_files) == 0) {

    stop("No TIF files found in ", input_dir)

}

cat("Found", length(tif_files), "files to convert.\n")

# Process each file

for (file in tif_files) {

    tryCatch(

        {

            # Read raster

            r <- rast(file)

            # Get base name for output

            file_name <- basename(file)

            file_name_no_ext <- tools::file_path_sans_ext(file_name)

            output_file <- file.path(output_dir, paste0(file_name_no_ext, ".asc"))

            cat("Converting:", file_name, "->", basename(output_file), "...\n")

            # Write to ASC

            # NAflag = -9999 ensures NoData is written as -9999

            writeRaster(r, output_file, filetype = "AAIGrid", NAflag = -9999, overwrite = TRUE)

        },

        error = function(e) {

            cat("Error converting", file, ":", e$message, "\n")

        }

    )

}

cat("All conversions completed.\n")

Optimization of Model Complexity Using ENMeval

# =============================================================================

# ENMeval MaxEnt Tuning Script for Bactrocera zonata (TERRA version)

# Species Distribution Modeling — Arabian Peninsula

# =============================================================================

# --- Allocate RAM to Java (MUST be set BEFORE loading rJava/dismo/ENMeval) ---

options(java.parameters = "-Xmx10g")   # 10 GB out of 16 GB total

# --- Load required libraries -------------------------------------------------

required_packages <- c("ENMeval", "terra", "dismo", "rJava",

                        "ggplot2", "dplyr", "knitr")

for (pkg in required_packages) {

  if (!requireNamespace(pkg, quietly = TRUE)) {

    install.packages(pkg, repos = "https://cran.r-project.org")

  }

  library(pkg, character.only = TRUE)

}

# Explicitly initialize the JVM

rJava::.jinit()

cat("All packages loaded successfully.\n")

cat("Java memory settings applied (10GB).\n")

# Check if maxent.jar is available

jar_path <- paste0(system.file("java", package="dismo"), "/maxent.jar")

if (!file.exists(jar_path)) {

  cat("WARNING: maxent.jar not found at", jar_path, "\n")

  cat("Please download 'maxent.jar' (from https://biodiversityinformatics.amnh.org/open_source/maxent/)")

  cat(" and place it in the dismo/java folder.\n")

} else {

  cat("maxent.jar found and ready.\n")

}

# --- Set paths ---------------------------------------------------------------

layers_path <- "C:/Users/solim/Desktop/AP/Bactrocera_zonata/Layers/Current"

occ_path    <- "C:/Users/solim/Desktop/AP/Bactrocera_zonata/occ.csv"

output_dir  <- "C:/Users/solim/Desktop/AP/Bactrocera_zonata/Results/ENMeval"

dir.create(output_dir, recursive = TRUE, showWarnings = FALSE)

# --- Load environmental layers with terra ------------------------------------

cat("\n--- Loading environmental layers with terra ---\n")

asc_files <- list.files(layers_path, pattern = "\\.asc$", full.names = TRUE)

cat("Found", length(asc_files), "layers:\n")

envs <- terra::rast(asc_files)

cat("Raster dimensions (nrow, ncol, nlyr):", nrow(envs), ",", ncol(envs), ",", nlyr(envs), "\n")

cat("Resolution:", res(envs), "\n")

cat("Extent:", as.character(ext(envs)), "\n")

# --- Load occurrence data ----------------------------------------------------

cat("\n--- Loading occurrence data ---\n")

occ_raw <- read.csv(occ_path, stringsAsFactors = FALSE)

cat("Total records loaded:", nrow(occ_raw), "\n")

# Extract lon/lat (columns 2 and 3)

occs <- occ_raw[, c("Longitude", "Latitude")]

colnames(occs) <- c("x", "y") # Use standard x, y for terra

# Remove any records with NA coordinates

occs <- occs[complete.cases(occs), ]

cat("Records after NA removal:", nrow(occs), "\n")

# Remove duplicates falling in the same raster cell

cells <- terra::cellFromXY(envs, as.matrix(occs))

dups  <- duplicated(cells)

if (any(dups)) {

  cat("Removing", sum(dups), "records in duplicate raster cells.\n")

  occs <- occs[!dups, ]

}

cat("Final occurrence records for modeling:", nrow(occs), "\n")

# Verify all points fall within the raster extent

extracted_vals <- terra::extract(envs, as.matrix(occs))

if("ID" %in% colnames(extracted_vals)) extracted_vals <- extracted_vals[, -1, drop=FALSE]

na_rows <- apply(extracted_vals, 1, function(x) any(is.na(x)))

if (any(na_rows)) {

  cat("WARNING: Removing", sum(na_rows), "points outside raster coverage.\n")

  occs <- occs[!na_rows, ]

}

cat("Valid occurrence records:", nrow(occs), "\n\n")

# --- Define tuning parameters ------------------------------------------------

fc_values <- c("L", "LQ", "H", "LQH", "LQHP")

rm_values <- seq(0.5, 4.0, by = 0.5)

cat("--- Tuning parameter grid ---\n")

cat("Feature classes:", paste(fc_values, collapse = ", "), "\n")

cat("Regularization multipliers:", paste(rm_values, collapse = ", "), "\n")

cat("Total combinations:", length(fc_values) * length(rm_values), "\n\n")

# --- Run ENMeval -------------------------------------------------------------

cat("=== Starting ENMeval (this may take a while) ===\n")

cat("Algorithm: maxent.jar\n")

cat("Start time:", format(Sys.time(), "%Y-%m-%d %H:%M:%S"), "\n\n")

# Sampling background points explicitly (10,000 pts)

bg_raw <- terra::spatSample(envs, 10000, "random", na.rm = TRUE, xy = TRUE)

bg <- bg_raw[, 1:2]

colnames(bg) <- c("x", "y")

cat("Occurrences head:\n")

print(head(occs))

cat("Background head:\n")

print(head(bg))

enmeval_results <- ENMevaluate(

  occs      = as.data.frame(occs),

  envs      = envs,

  bg        = as.data.frame(bg),

  algorithm = "maxent.jar",

  partitions = "block",

  tune.args  = list(fc = fc_values, rm = rm_values),

  parallel   = FALSE,

  doClamp    = TRUE

)

cat("\nEnd time:", format(Sys.time(), "%Y-%m-%d %H:%M:%S"), "\n")

cat("=== ENMeval completed successfully! ===\n\n")

# --- Extract and save results ------------------------------------------------

cat("\n--- Extracting results ---\n")

results <- eval.results(enmeval_results)

if (is.null(results) || nrow(results) == 0) {

  stop("No results found in ENMeval object.")

}

# Sort by AICc (primary criterion)

results_sorted <- results[order(results$delta.AICc), ]

# Save full results table

write.csv(results_sorted,

          file.path(output_dir, "enmeval_full_results.csv"),

          row.names = FALSE)

# --- Identify optimal model --------------------------------------------------

cat("--- Model Selection Summary ---\n\n")

best_aic <- results_sorted[1, ]

cat("Best model by AICc:\n")

cat("  Feature classes:", as.character(best_aic$fc), "\n")

cat("  Regularization :", as.character(best_aic$rm), "\n")

cat("  AICc           :", round(best_aic$AICc, 2), "\n")

cat("  AUC.train      :", round(best_aic$auc.train, 4), "\n")

cat("  AUC.val (mean) :", round(best_aic$auc.val.avg, 4), "\n\n")

# --- Generate diagnostic plots -----------------------------------------------

cat("--- Generating diagnostic plots ---\n")

try({

  p1 <- ggplot(results, aes(x = as.numeric(rm), y = AICc, color = fc, group = fc)) +

    geom_line(linewidth = 1) + geom_point(size = 2.5) + theme_minimal() +

    labs(title = "AICc Analysis", x = "Regularization Multiplier", y = "AICc")

  ggsave(file.path(output_dir, "plot_AICc.png"), p1, width = 8, height = 5)

  p2 <- ggplot(results, aes(x = as.numeric(rm), y = auc.val.avg, color = fc, group = fc)) +

    geom_line(linewidth = 1) + geom_point(size = 2.5) + theme_minimal() +

    labs(title = "Validation AUC", x = "Regularization Multiplier", y = "Mean AUC")

  ggsave(file.path(output_dir, "plot_AUC.png"), p2, width = 8, height = 5)

}, silent = FALSE)

# --- Save best model prediction map ------------------------------------------

cat("\n--- Saving optimal model prediction raster ---\n")

best_idx <- which(results$delta.AICc == 0)[1]

preds <- eval.predictions(enmeval_results)

if (!is.null(preds)) {

  best_pred <- preds[[best_idx]]

  terra::writeRaster(best_pred,

                     file.path(output_dir, "best_model_prediction.tif"),

                     overwrite = TRUE)

  # Plot manually

  try({

    png(file.path(output_dir, "best_model_prediction_map.png"), width = 2000, height = 1500, res = 300)

    terra::plot(best_pred, main = paste0("Best Model (FC=", best_aic$fc, ", RM=", best_aic$rm, ")"))

    points(occs$longitude, occs$latitude, pch = 16, cex = 0.5, col = "red")

    dev.off()

  })

}

# Save the object

saveRDS(enmeval_results, file.path(output_dir, "enmeval_object.rds"))

cat("\n=====================================================\n")

cat("  ENMeval TUNING COMPLETE (TERRA VERSION)\n")

cat("  Results saved to:", output_dir, "\n")

cat("=====================================================\n")
