# Supplementary material for: Climatic Niche Contraction and Refugial Persistence of an Invasive Tephritid Pest Across the Arabian Peninsula Under Contrasting Emission Scenarios
Source: Biology (Basel). 2026 May 21;15(10):814. doi: 10.3390/biology15100814 (PMC13203219; doi:10.3390/biology15100814)
Supplement: Supplementary file 1 [file biology-15-00814-s001.zip › Figure S1.pdf]

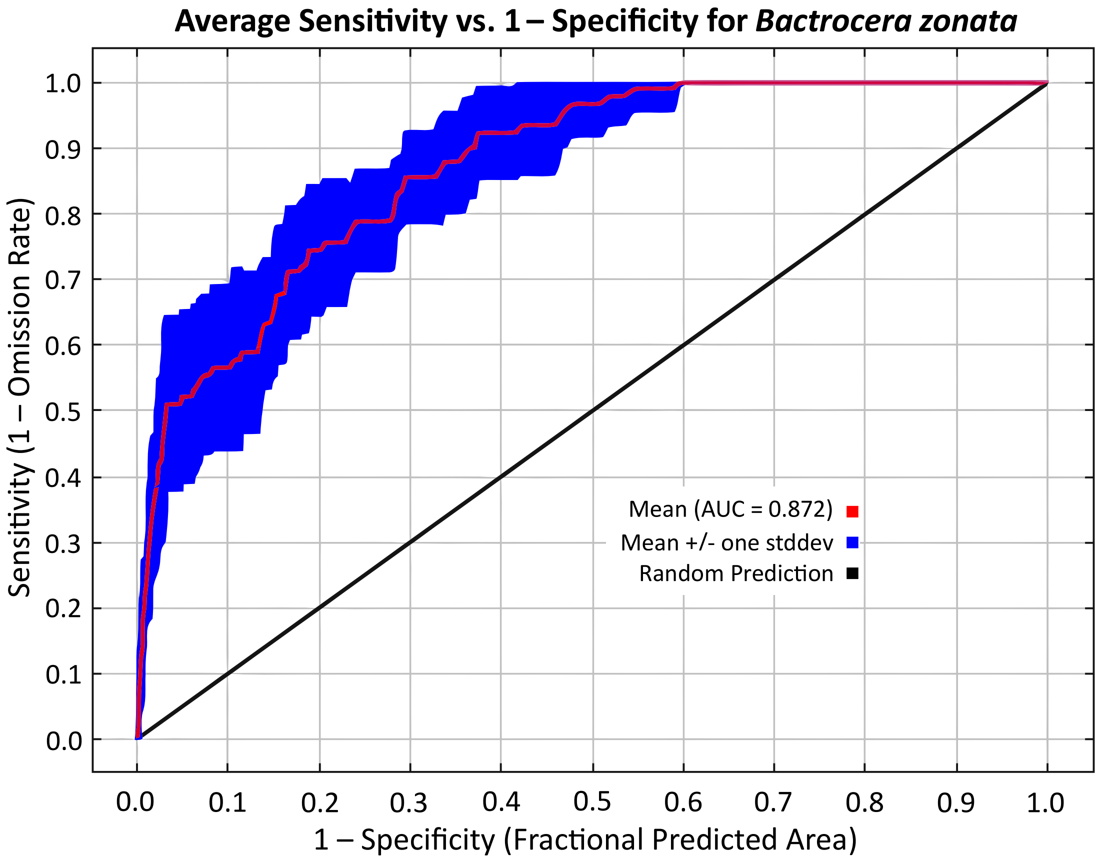

Figure S1. Receiver Operating Characteristic (ROC) curve for the MaxEnt species distribution model of *Bactrocera zonata* across the Arabian Peninsula. The red line represents the mean sensitivity (1 − omission rate) against 1 − specificity (fractional predicted area) across replicate model runs, with blue shading indicating ± one standard deviation.
