# Supplementary material for: Climatic Niche Contraction and Refugial Persistence of an Invasive Tephritid Pest Across the Arabian Peninsula Under Contrasting Emission Scenarios
Source: Biology (Basel). 2026 May 21;15(10):814. doi: 10.3390/biology15100814 (PMC13203219; doi:10.3390/biology15100814)
Supplement: Supplementary file 1 [file biology-15-00814-s001.zip › Figure S2.pdf]

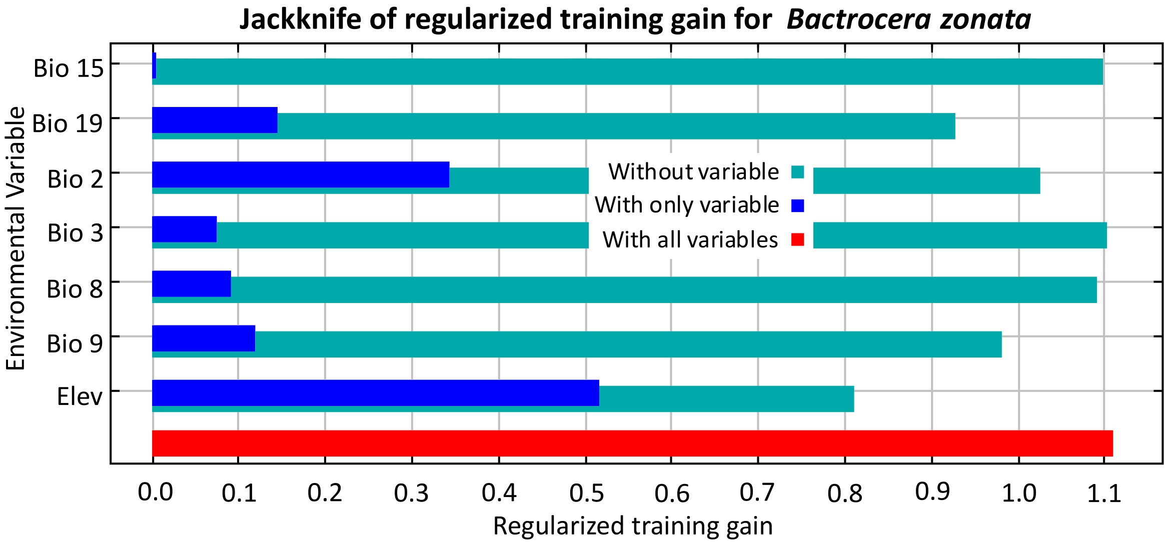

Figure S2. Jackknife analysis of regularized training gain for *Bactrocera zonata* MaxEnt habitat suitability model, illustrating the relative importance of each environmental predictor. Teal bars indicate model training gain when the respective variable is withheld; blue bars represent gain when only that variable is used in isolation; and the red bar denotes the gain of the full model using all variables.
