# Supplementary material for: Climatic Niche Contraction and Refugial Persistence of an Invasive Tephritid Pest Across the Arabian Peninsula Under Contrasting Emission Scenarios
Source: Biology (Basel). 2026 May 21;15(10):814. doi: 10.3390/biology15100814 (PMC13203219; doi:10.3390/biology15100814)
Supplement: Supplementary file 1 [file biology-15-00814-s001.zip › File S5.docx]

**S5 File.** Details of the primary five General Circulation Climate Models (GCMs) used for the prediction of future habitat suitability of *Bactrocera zonata*.

| Model ID | Institution | native  resolution | ensemble  member | priority^a^ |
| --- | --- | --- | --- | --- |
| GFDL-ESM4 | National Oceanic and Atmospheric  Administration, Geophysical Fluid  Dynamics Laboratory, USA | 288x180 | r1i1p1f1 | 1 |
| IPSL-CM6A-LR | Institute Pierre Simon Laplace, France | 144x143 | r1i1p1f1 | 4 |
| MPI-ESM1-2-HR | Max Planck Institute for Meteorology, Germany | 384x192 | r1i1p1f1 | 3 |
| MRI-ESM2-0 | Meteorological Research Institute, Japan | 320x160 | r1i1p1f1 | 5 |
| UKESM1-0-LL | Met Office Hadley Centre, UK | 192x144 | r1i1p1f2 | 2 |

^a^The priority of models follows the suggestions of the ISIMIP3b protocol.
