# Supplementary material for: Climatic Niche Contraction and Refugial Persistence of an Invasive Tephritid Pest Across the Arabian Peninsula Under Contrasting Emission Scenarios
Source: Biology (Basel). 2026 May 21;15(10):814. doi: 10.3390/biology15100814 (PMC13203219; doi:10.3390/biology15100814)
Supplement: Supplementary file 1 [file biology-15-00814-s001.zip › File S4.docx]

**S4 File.** Variance Inflation Factor (VIF) analysis of environmental variables used in the distribution modeling of *Bactrocera zonata* in the Arabian Peninsula. Variables with VIF > 5 were iteratively removed.

| **Variable** | **Description** | **VIF (Before Selection)** | **VIF (After Selection)** | **Status** |
| --- | --- | --- | --- | --- |
| Bio1 | Annual Mean Temperature | 10,086.60 | — | Excluded |
| Bio2 | Mean Diurnal Range | 1,001.31 | 1.25 | Retained |
| Bio3 | Isothermality | 699.66 | 3.51 | Retained |
| Bio4 | Temperature Seasonality | 7,981.34 | — | Excluded |
| Bio5 | Max Temperature of Warmest Month | 120,095,990,063,213.23 | — | Excluded |
| Bio6 | Min Temperature of Coldest Month | 93,824,992,236,885.33 | — | Excluded |
| Bio7 | Temperature Annual Range | 155,296,538,874,844.69 | — | Excluded |
| Bio8 | Mean Temperature of Wettest Quarter | 24.85 | 2.09 | Retained |
| Bio9 | Mean Temperature of Driest Quarter | 24.09 | 4.04 | Retained |
| Bio10 | Mean Temperature of Warmest Quarter | 5,530.40 | — | Excluded |
| Bio11 | Mean Temperature of Coldest Quarter | 15,086.48 | — | Excluded |
| Bio12 | Annual Precipitation | 1,666.18 | — | Excluded |
| Bio13 | Precipitation of Wettest Month | 84.42 | — | Excluded |
| Bio14 | Precipitation of Driest Month | 104.54 | — | Excluded |
| Bio15 | Precipitation Seasonality | 72.87 | 1.6 | Retained |
| Bio16 | Precipitation of Wettest Quarter | 851.72 | — | Excluded |
| Bio17 | Precipitation of Driest Quarter | 106.97 | — | Excluded |
| Bio18 | Precipitation of Warmest Quarter | 198.35 | — | Excluded |
| Bio19 | Precipitation of Coldest Quarter | 94.40 | 1.7 | Retained |
| Elev | Elevation | 124.41 | 4.12 | Retained |
| *VIF threshold = 5. Variables with VIF > 5 were iteratively excluded using the vifstep function from the usdm package in R.* | | | | |
