# Supplementary material for: Climatic Niche Contraction and Refugial Persistence of an Invasive Tephritid Pest Across the Arabian Peninsula Under Contrasting Emission Scenarios
Source: Biology (Basel). 2026 May 21;15(10):814. doi: 10.3390/biology15100814 (PMC13203219; doi:10.3390/biology15100814)
Supplement: Supplementary file 1 [file biology-15-00814-s001.zip › File S7.docx]

**S7 File.** Performance and Evaluation of the MaxEnt model using Area Under the ROC curve (AUC) and True Skilled Statistics (TSS).

| Model replicate | AUC^1^ | | True Skill Statistic (TSS)^2^ |
| --- | --- | --- | --- |
|  | Training | Test |  |
| 1 | 0.909 | 0.879 | 0.689 |
| 2 | 0.913 | 0.912 | 0.656 |
| 3 | 0.920 | 0.907 | 0.692 |
| 4 | 0.911 | 0.878 | 0.473 |
| 5 | 0.933 | 0.845 | 0.368 |
| 6 | 0.933 | 0.863 | 0.492 |
| 7 | 0.914 | 0.886 | 0.480 |
| 8 | 0.944 | 0.817 | 0.400 |
| 9 | 0.932 | 0.845 | 0.549 |
| 10 | 0.916 | 0.880 | 0.581 |
| Average ± SD | 0.922 ± 0.011 | 0.872 ± 0.029 | 0.538 ± 0.115 |

^1^The area under the curve (AUC) has scores varying between 0 and 1, and values ≥0.7 are considered acceptable.

^2^True skilled statistics (TSS) scores vary from -1 to 1, and values ≥0.5 indicate acceptable models.
