# Supplementary material for: Climatic Niche Contraction and Refugial Persistence of an Invasive Tephritid Pest Across the Arabian Peninsula Under Contrasting Emission Scenarios
Source: Biology (Basel). 2026 May 21;15(10):814. doi: 10.3390/biology15100814 (PMC13203219; doi:10.3390/biology15100814)
Supplement: Supplementary file 1 [file biology-15-00814-s001.zip › File S8.docx]

**S8 File.** Projected current and future suitable habitat extent (km² and percentage of total area) for *Bactrocera zonata* under Shared Socioeconomic Pathway scenarios SSP1-2.6 and SSP5-8.5 for the 2050- and 2070-time horizons, incorporating quantitative assessments of proportional range expansion and contraction relative to the contemporary baseline distribution.

| **Time Period** | **Current Distribution** | | **SSP1-2.6** | | | | **SSP5-8.5** | | | |
| --- | --- | --- | --- | --- | --- | --- | --- | --- | --- | --- |
|  | **Area (km²)** | **Area (%)** | **Area (km²)** | **Area (%)** | **Expansion (%)** | **Contraction (%)** | **Area (km²)** | **Area (%)** | **Expansion (%)** | **Contraction (%)** |
| 2050 | 790,714 | 28.38 | 732,376 | 26.28 | 12.48 | 19.85 | 625,339 | 22.44 | 3.76 | 24.67 |
| 2070 |  |  | 722,700 | 25.94 | 16.44 | 25.05 | 569,265 | 20.43 | 3.79 | 31.80 |
